# Targeting the antioxidant, antimicrobial and anti-inflammatory activity of non-psychotropic *Cannabis sativa* L.: a comparison with chemotype V

**DOI:** 10.1186/s42238-025-00336-1

**Published:** 2025-10-21

**Authors:** Chiara Ceresa, Martina Delsignore, Matej Maly, Francesca Carrà, František Beneš, Andrea Chiara Sansotera, Aurora Camola, Marco Arlorio, Chiara Porta, Letizia Fracchia, Vincenzo Disca, Federica Pollastro

**Affiliations:** 1 Department of Pharmaceutical Sciences, University of Piemonte Orientale, L.go Donegani 2, Novara, 28100 Italy; 2 Department of Food Analysis and Nutrition, University of Chemistry and Technology in Prague, Technická 5, Prague 6, 166 28 Czech Republic; 3 Center for Translational Research on Autoimmune & Allergic Diseases (CAAD), University of Piemonte Orientale, C.so Trieste 15/A, Novara, 28100 Italy

**Keywords:** Cannabis chemotypes, Cannabichromene, Cannabinoid extracts, Chemotype v

## Abstract

**Background:**

Non-psychotropic *Cannabis sativa* L. chemotypes have gained increasing interest due to their diverse profiles of bioactive compounds. While cannabinoids such as cannabidiol (CBD), cannabigerol (CBG), are known for their biological effects, the role of other cannabinoids such cannabichromene (CBC) remains underexplored as for chemotype V, which lacks in cannabinoids but is characterized by other minor phytochemicals.

**Objective:**

This study aimed to evaluate the individual and combined contributions of cannabinoids and non-cannabinoid phenolics to the antioxidant, antimicrobial, and anti-inflammatory properties of extracts derived from four *C. sativa* chemotypes, including a cannabinoid-free variant as a comparison.

**Methods:**

Ethanolic extracts were obtained from four hemp chemotypes: CBD-rich (CS1), CBG-rich (CS2), CBC-rich (CS3), and cannabinoid-free (CS4). Phytochemical profiling was conducted using UHPLC-HRMS. Antioxidant properties were assessed via DPPH, ABTS, and FRAP assays. Antimicrobial activity was tested against Gram-positive and Gram-negative bacteria through MIC, MBC, and time-kill assays. Anti-inflammatory activity was evaluated in LPS-stimulated RAW 264.7 macrophages via gene expression analysis of pro- and anti-inflammatory mediators (IL1b, IL6, Cox2, IL10, IL1Ra).

**Results:**

Phytochemical analysis confirmed the chemotype-specific profiles, with CS3 showing the highest levels of canniprene and the early discovered 5-methoxy-dihydrodenbinobin. Antioxidant assays revealed that cannabinoids were the main contributors to radical scavenging capacity, though CS3 exhibited additional ferric ion reducing power likely due to non-cannabinoid phenolics. Antibacterial activity was confined to Gram-positive bacteria, where CS1 showed the highest efficacy, and CS4 showed no activity, highlighting the critical role of cannabinoids. All extracts reduced LPS-induced *Il1b*, *Il6*, and *Cox2* gene expression, but only cannabinoid-rich extracts upregulated the anti-inflammatory cytokines IL10 and IL1Ra, indicating a cannabinoid-dependent effect.

**Conclusion:**

Both cannabinoids and non-cannabinoid phenolics contribute to the biological activity of *Cannabis sativa* extracts, with cannabinoids playing a central role in antimicrobial responses and stronger anti-inflammatory effect as a pure cannabinoid or as an extract. From this point of view, the cannabinoid-free chemotype V could be a valuable functional control for isolating the effects of cannabinoids, reinforcing the need for integrative analyses in evaluating the therapeutic potential of cannabis-derived formulations.

**Supplementary Information:**

The online version contains supplementary material available at 10.1186/s42238-025-00336-1.

## Introduction

*Cannabis sativa* L. is one of the most versatile plants, with a wide range of heterogeneous applications spanning from the pharmaceutical to the industrial sectors. In recent decades, cannabis has gained renewed attention due to advancements in harvesting techniques and breeding, which have led to the development of different, well-established chemotypes within the genus, classified based on the concentrations of major cannabinoids. Among these chemotypes, the primary non-psychoactive types that characterize industrial hemp include: (III) fiber hemp, where cannabidiol (CBD) is the predominant constituent and Δ^9^-THC content is below 0.2% w/w; (IV) fiber-type plants dominated by cannabigerol (CBG); and (V) fiber-type plants that are largely devoid of cannabinoids (Borroto Fernandez et al. 2020; de Meijer, E.P., 2014).

Although CBD accounts for more than half of the total cannabis market, the progressive liberalization of hemp cultivation and ongoing phytochemical research have sparked growing interest in other cultivars. Notably, there is an increasing focus on type IV, which contains CBG, as well as the latest developed cannabichromene (CBC) chemotype (de Meijer et al. 2009; de Meijer 2014). Although these chemotypes are gaining attention due to their significant meroterpenoid properties as antioxidant, antimicrobial, and anti-inflammatory effects (Aqawi et al. 2021; Fiorentino et al. 2024; Koltai and Namdar 2020), research into the biological targets of CBG and CBC is still in its early stages compared to CBD, highlighting the need for further studies (Gojani et al. 2023).

Additionally, the growing public awareness of cannabinoids has led to the development of various green extraction methods for cannabis inflorescences, making it possible to use both pure cannabinoids as active ingredients and cannabinoid-rich extracts (Valizadehderakhshan et al. 2021).

Chemotype V, a cannabinoid-free variety of *Cannabis sativa* L., provides a unique model system for investigating the biological activity of different strains of cannabis. Although frequently overlooked due to the almost total absence of cannabinoids, its inclusion in experimental designs allows for the controlled assessment of cannabinoid contribution to extract activity, supports sensory blinding protocols, and facilitates the study of potential interactions among cannabis metabolites (Russo 2019; Salamone et al. 2022).

In the present study, we capitalize on the phytochemical characterization of four samples of cannabis chemotypes evaluating the biological activity of each phytocomplex highlighting the role played in this contest by the major cannabinoid characterizing each chemotype, particularly in relation to the cannabinoid-free type.

Given the well-established role of skin dysbiosis in exacerbating inflammatory skin diseases—such as atopic dermatitis, psoriasis, rosacea, and acne—there is growing therapeutic interest in identifying bioactive compounds that can restore microbial balance and reduce inflammation. In this context, we evaluated the biological activity of cannabis extracts in comparison to pure cannabinoids and chemotype V. Specifically, we assessed their antioxidant activity, the antimicrobial effect against a panel of Gram-positive and Gram-negative microorganisms, and together with the anti-inflammatory properties using lipopolysaccharides (LPS)-activated macrophages.

## Materials and methods

Experimental procedures employed standard chromatographic techniques, including low-pressure liquid chromatography, HPLC, and TLC, as well as NMR, UV–vis spectrophotometry, and UHPLC-HRMS for compound characterization and quantification. *Cannabis sativa* inflorescences from different chemotypes (CBD-, CBG-, CBC-rich, and a no-cannabinoid type) were extracted with ethanol by maceration. Selected material underwent further purification, decarboxylation, and chromatographic separation to isolate CBD, CBG, and CBC, which were identified by NMR. Antioxidant activity of crude extracts and purified cannabinoids was evaluated using DPPH•, ABTS•+, and FRAP assays, with results expressed as Trolox equivalents. Phytochemical profiling was carried out by UHPLC-HRMS, enabling the quantification of cannabinoids and non-cannabinoid phenolics against authentic standards.

The antibacterial activity of extracts, isolated cannabinoids, and reference antibiotics was assessed against a panel of Gram-positive and Gram-negative strains, including MRSA, through broth microdilution assays to determine MICs, MBCs, and time–kill kinetics. In parallel, murine RAW 264.7 macrophages were used to evaluate cytotoxicity by AlamarBlue™ and to investigate the anti-inflammatory potential of extracts. Cells were pre-treated with non-cytotoxic concentrations and stimulated with LPS, followed by RNA extraction, cDNA synthesis, and qPCR analysis of inflammatory gene expression. Data were analyzed using ANOVA followed by Tukey’s post-hoc test, with significance set at *p* < 0.05.

All experimental details, including instrumentation, bacterial and cell culture conditions, extraction protocols, and analytical procedures, are provided in full in the Supplementary Materials.

## Results

### Cannabis samples and extraction yields

Ethanolic extractions were performed on the four different chemotypes of hemp samples (Table 1). Among the four samples, CS1 exhibited the highest extraction yield at 6.78%, followed by CS2 and CS3, with intermediate yields of 4.18, 3.53, and 3.17%, respectively. CS4, designed to be free from cannabinoids, displayed the lowest yield (2.93%).

**Table 1:** Sample coding and extraction yields expressed as percentage of the ethanolic extract mass to the homogenized hemp mass

Samples	Chemotypes	Extraction yield (%)
CS1	III- Industrial fiber hemp with CBD as predominant and a minimum content of ∆^9^-THC (from 0.2% w/w to 0.6% w/w)	6.78
CS2	IV- Industrial fiber hemp with CBG as predominant cannabinoid	4.18
CS3	Chemotype with CBC as major cannabinoid	3.17
CS4	V- Industrial fiber hemp with almost no cannabinoids	2.93

### Quantitative analysis of cannabinoids and non-cannabinoid phenolic compounds

**Fig. 1:**
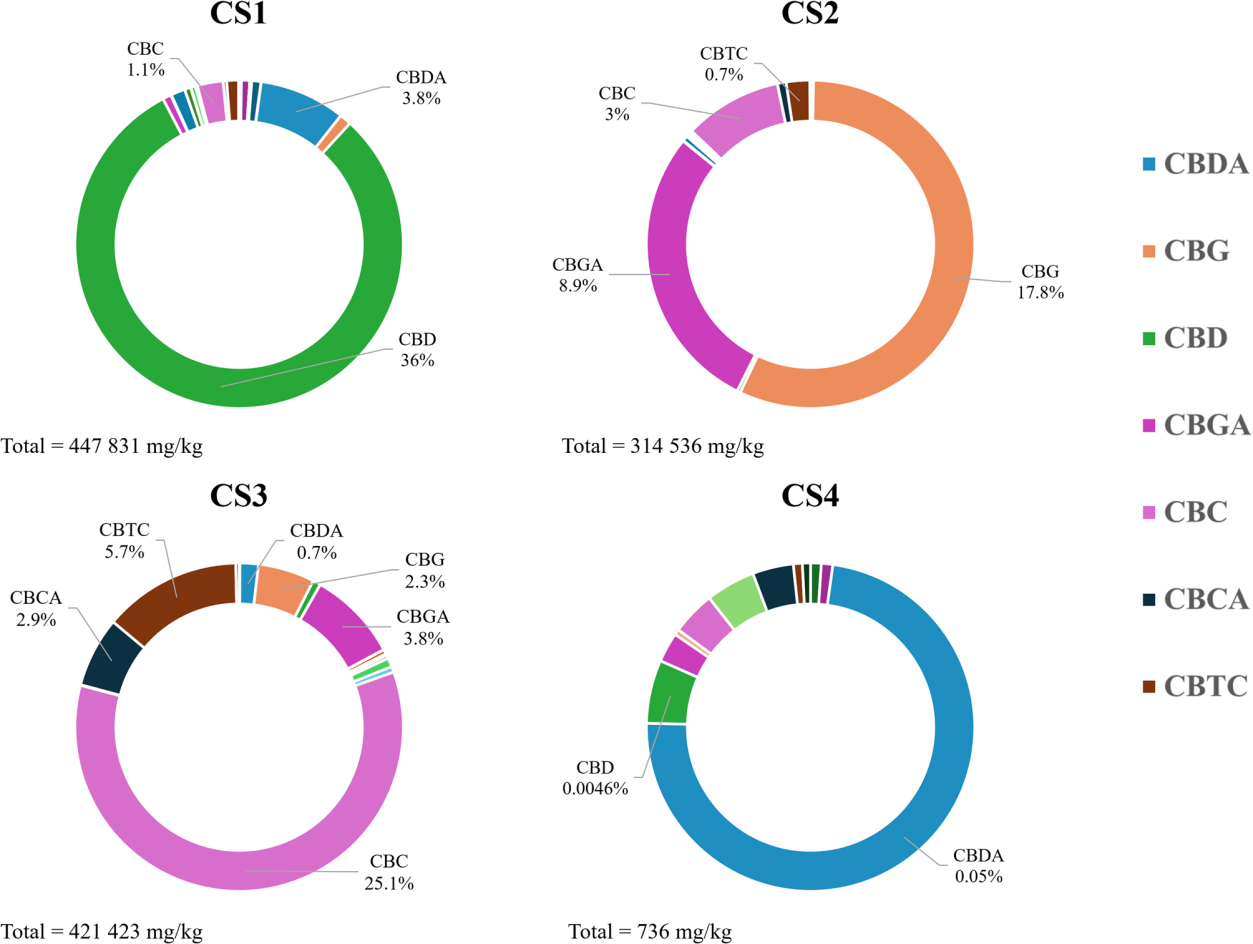
Major cannabinoids content and the total cannabinoids content in each extract expressed as a percentage over crude extract weight

The UHPLC-HRMS analysis was performed on non-decarboxylated samples. The percentage of the identified cannabinoids present in each extract are shown in Fig. 1, while concentration expressed in mg/kg is reported in Table S2 of the Supporting Information and reflects the classification in chemotypes. ∆^9^-THC/∆^9^-THCA are present in all the strains, with concentrations ranging from a minimum of 35.9 to a maximum of 3660 mg/kg in CS3, which presents the highest concentration of cannabicitran 4 (CBTC) with a content of 57,305 mg/kg. Minor cannabinoids with modification in the polyketide clusters as propyl- and butyl-homolog are also detectable. As expected, the CS4 extract contains an almost undetectable concentration of cannabinoids (0.07%).

Regarding the quantification of the other constituents, beside the common flavonoids, we took in consideration non-cannabinoid phenolic compounds recognised as unique of *Cannabis sativa*: as canniprene, a bibenzyl able to inhibit the production of inflammatory eicosanoids via the 5-lipoxygenase (5-LOX) pathway (Allegrone et al. 2017), the prenylated flavonoids cannflavin A and B, known for their inhibition of the microsomal prostaglandin E2 synthase (mPGES)-1 (Werz et al. 2014), and, for the first time, the 5-methoxy-dihydrodenbinobin, a dihydrophenanthrene recently discovered in chemotype V, involved in the suppression of the pro-inflammatory leukotriene biosynthesis in activated macrophage subtypes by targeting 5-LOX (Salamone et al. 2022).

**Fig. 2:**
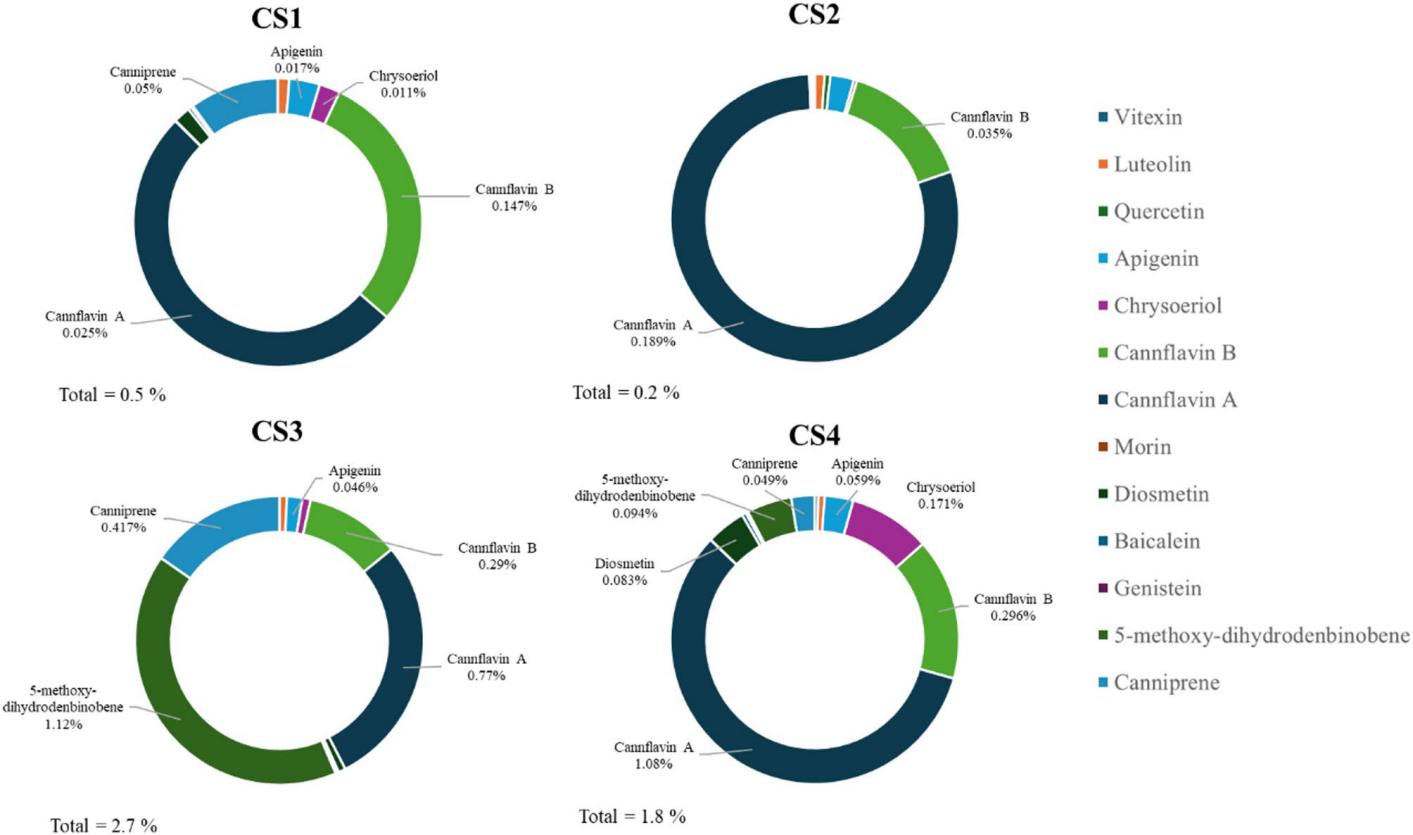
Major non-cannabinoid compounds content and the total amount in the extracts expressed as a percentage over crude extract weight

These latter compounds together with the other flavonoids are present in all the samples analyzed with a concentration ranging from 0.05 to a maximum of 2.73% (Fig. 2 and Table S3 in Supporting Information).

The results evidenced that, concerning flavonoids, all the different chemotypes are dominated by the presence of cannflavin A and B (Fig. 2). Despite CS4 containing canniprene (491 mg/kg) and 5-methoxy-dihydrodenbinobin (940 mg/kg) in a valuable concentration, these metabolites are more abundantly biosynthesized in CS3 respectively with a concentration of 11,168 and 4 168 mg/kg (Table 2).

**Table 2:** Concentration of Cannflavin A, canniprene and 5-methoxy-dihydrodenbinobin expressed as mg/kg in samples CS1, CS2, CS3 and CS4

Compound (mg/kg)	CS1	CS2	CS3	CS4
Cannflavin A	2558 ± 91^c^	1892 ± 67^d^	7709 ± 62^b^	10,803 ± 12^a^
Canniprene	502 ± 21^b^	< LOQ	4168 ± 337^a^	491 ± 16^b^
5-methoxy-dihydrodenbinobine	< LOQ	< LOQ	11,168 ± 74^a^	940 ± 108^b^

Different letters indicate statistical differences (*p* < 0.05), within the same molecule.

### Antioxidant activity

The antioxidant activity determined with DPPH, ABTS, and FRAP assays, was evaluated for each cannabis extract and compared to the pure cannabinoids (Table 3).

**Table 3:** DPPH, ABTS and FRAP assay results are reported as mean ± standard deviation (*n* = 3). Values are expressed as g of trolox equivalents per kg of sample

	CS1	CS2	CS3	CS4		CBD	CBG	CBC
DPPH	32.6 ± 1.7^aA^	30.9 ± 1.8^aB^	29.3 ± 0.3^bB^	19.4 ± 0.4^c^		29.2 ± 0.1^bB^	33.7 ± 0.9^aA^	32.9 ± 0.1^aA^
ABTS	830 ± 25^aA^	385 ± 12^bA^	290 ± 11^cA^	nd		321 ± 22^aB^	310 ± 7^aB^	230 ± 13^bB^
FRAP	41.3 ± 8^bcA^	51.4 ± 1.7^ab^	57.3 ± 1.7^a^	31.6 ± 1.7^c^		22.9 ± 0.6^B^	nd	nd

Different lowercase letters indicate statistical differences (*p* < 0.05) within pure molecule (CBD, CBG and CBC) and different extracts (CS1, CS2, CS3 and CS4) while different capital letters indicate statistical differences (*p* < 0.05) between the extract and the relative most abundant pure cannabinoid (CS1 vs. CBD, CS2 vs. CBG. CS4 vs. CBC). nd = not detected

In the DPPH assay, the highest antioxidant activity was observed in sample CS1 (32.6 g TE/kg), and the lowest in sample CS4 (19.4 g TE/kg). Samples CS2 (30.9 g TE/kg) and CS3 (29.3 g TE/kg) showed intermediate antioxidant activities, with CS3 being lower than CS2. Among the pure compounds, CBG exhibited the highest DPPH radical scavenging activity (33.7 g TE/kg), followed by CBC (32.9 g TE/kg) and CBD (29.2 g TE/kg). While CBC alone was more efficient than the relative extract CS3, CBG showed no difference compared to CS2 and CBD showed to have lower antioxidant activity than CS1. The ABTS assay revealed significant variation in antioxidant capacity among the samples. CS1 demonstrated the highest antioxidant activity (830 g TE/kg), significantly greater than CS2 (385 g TE/kg) and CS3 (290 g TE/kg). CS4 was non-detectable in this assay, likely due to the absence of cannabinoids. Among the pure compounds, CBD (321 g TE/kg) showed higher activity than CBG (310 g TE/kg) and CBC (230 g TE/kg). The FRAP assay, showed that CS3 exhibited the highest activity (57.3 TE/kg), followed by CS2 (51.4 g TE/kg) and CS1 (41.3 g TE/kg). CS4 demonstrated the lowest reducing power (31.6 g TE/kg). Among the pure compounds, CBD (22.9 g TE/kg) was the only one detectable in this assay, as CBG and CBC were not detected.

### Antimicrobial activity

#### MIC determination

The antibacterial activity of each extract and control antibiotics was determined against both Gram-positive and Gram-negative bacteria by MICs determination. Results showed that all the cannabinoid-rich extracts were able to affect the growth of Gram-positive bacteria. Specifically, CS1 showed the highest inhibitory activity, with MIC values ranging from 2.5 to 10 µg/mL, being particularly effective against MRSA (5 µg/mL), *Staphylococcus epidermidis* (5 µg/mL), and *Bacillus cereus* (2.5 µg/mL). In contrast, extract CS4 was completely inactive (MIC > 100 µg/mL) against all Gram-positive strains, while CS2 and CS3 demonstrated moderate activity compared to CS1, with MIC values between 10 and 20 µg/mL. The results obtained are consistent with previous findings reported in the literature (Aiemsaard et al. 2022), further confirming the efficacy of cannabinoids in inhibiting the growth of Gram-positive bacteria (Table 4).

**Table 4:** Minimum inhibitory concentration (MIC, µg/mL) of CS extracts and control antibiotics against Gram-positive bacteria

	Bacterial strain				
**Extract/drug**	***S. aureus***	***MRSA***	***S. epidermidis***	***B. cereus***	***L. monocytogenes***
CS1	10	5	5	2.5	10
CS2	20	20	10	10	10
CS3	20	20	10	10	10
CS4	> 100	> 100	> 100	> 100	> 100
tetracycline	0.5	0.5	> 2	> 2	0.5
ciprofloxacin	0.125	0.25	0.125	0.125	1
linezolid	2	2	0.5	1	2
methicillin	2	> 2	> 2	> 16	> 16

All the extracts were inactive against the two tested Gram-negative strains, *Escherichia coli* and *Salmonella enterica* (MIC > 100 µg/mL). Regarding the MIC of pure cannabinoids, CBD and CBG showed the highest antibacterial activity with a MIC = 2.5 µg/mL against all the Gram-positive bacteria tested strains. CBC exhibited the strongest antibacterial activity against *S. epidermidis* (MIC = 2.5 µg/mL), intermediate activity against *B. cereus* and *L. monocytogenes* (MIC = 5 µg/mL) and a less pronounced effect against both the two *S. aureus* and MRSA strains (MIC = 10 µg/mL).

Moreover, correlating the results obtained with the pure cannabinoids to their concentration in the respective chemotypes, MIC values against Gram-positive bacterial strains remained broadly consistent with those obtained using the extracts. This alignment, with only minor variations observed, strongly suggests that the antibacterial activity of the extracts is primarily attributable to their cannabinoid content, rather than other non-cannabinoid phenolic compounds.

#### MBC determination

To define whether CS1, CS2 and CS3 chemotypes possessed a bacteriostatic or bactericidal activity, the MBCs (representing the lowest extract concentration required to kill 99.9% of the bacterial population), were determined (Table 5). The MBCs values of the three extracts overlapped with the corresponding MICs for *S. aureus*, MRSA and *B. cereus*, suggesting a marked bactericidal activity against these strains. Concerning *S. epidermidis* and *L. monocytogenes* the values of MBCs were equal or slightly higher than the MICs, indicating a more moderate bactericidal effect against these Gram-positive bacteria.

**Table 5:** Minimum bactericidal concentration (MBC, µg/mL) of CS extracts against Gram-positive bacteria

	Bacterial strain				
**Extract/drug**	***S. aureus***	***MRSA***	***S. epidermidis***	***B. cereus***	***L. monocytogenes***
CS1	10	5	10	2.5	40
CS2	20	20	20	10	20
CS3	20	20	10	10	20

#### Time-kill

**Fig. 3:**
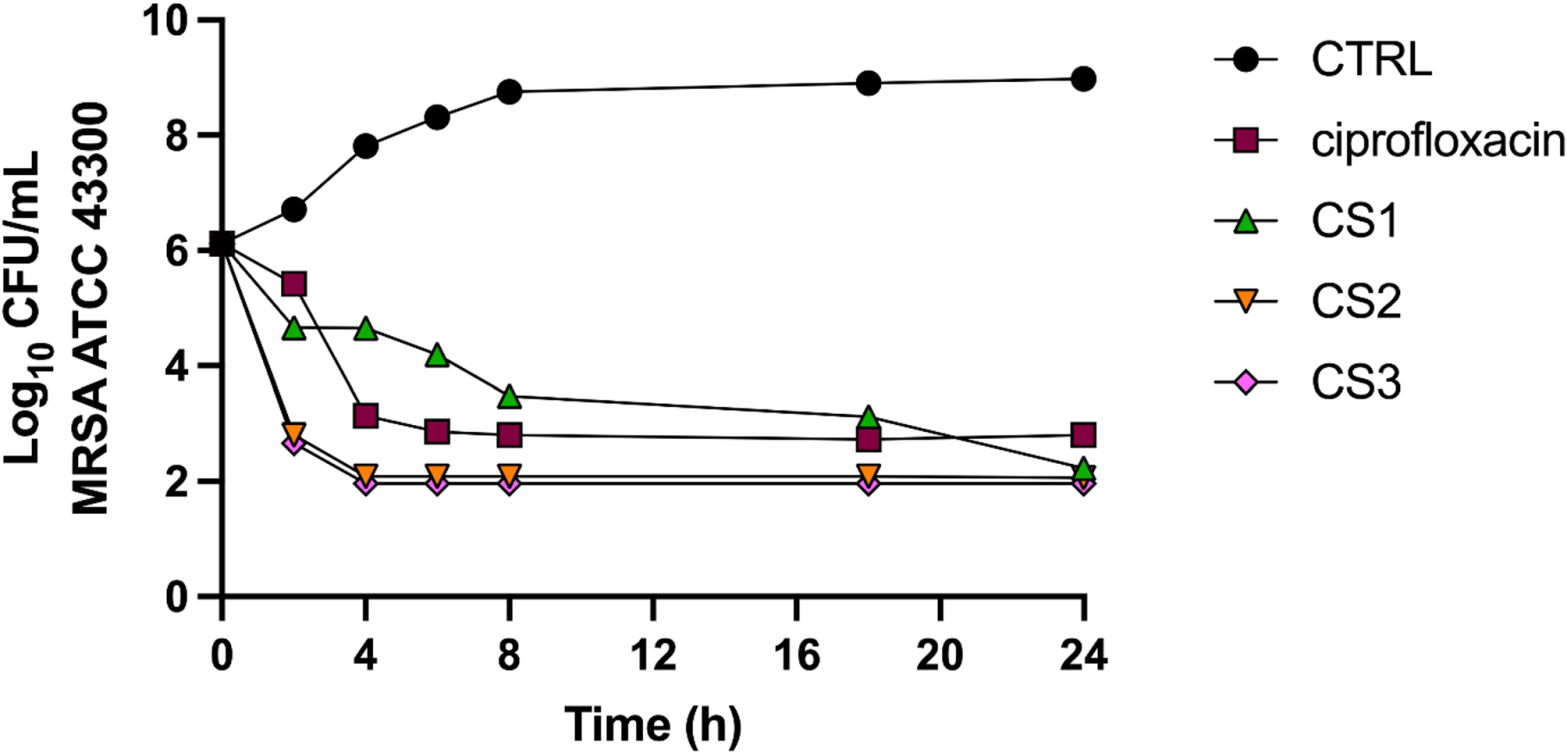
Time–kill analysis of CS extracts CS1, CS2, CS3 and ciprofloxacin at 4 × MIC against methicillin-resistant *S. aureus* (MRSA) ATCC 43,300 over a 24 h incubation period at 37 °C

To support the MBC results and gain deeper insight into the time-course of bacterial reduction by the CS1-3 extracts, a time-kill assay was conducted against the methicillin-resistant *S. aureus* strain. A ≥ 3-log decrease (≥ 99.9%) in MRSA CFU/mL within 24 h is indicative of bactericidal activity. Overall, like the control bactericidal agent ciprofloxacin, all the tested extracts significantly reduced the number of bacterial cells by 3-log or more. The results showed that CS2 and CS3 exhibited a more pronounced bactericidal effect, which occurred within 2 h and remained stable over time (Fig. 3). Regarding CS1, the bacterial population gradually declined over time, reaching a 3-log reduction within 18 h.

### Evaluation of murine macrophage viability

**Fig. 4:**
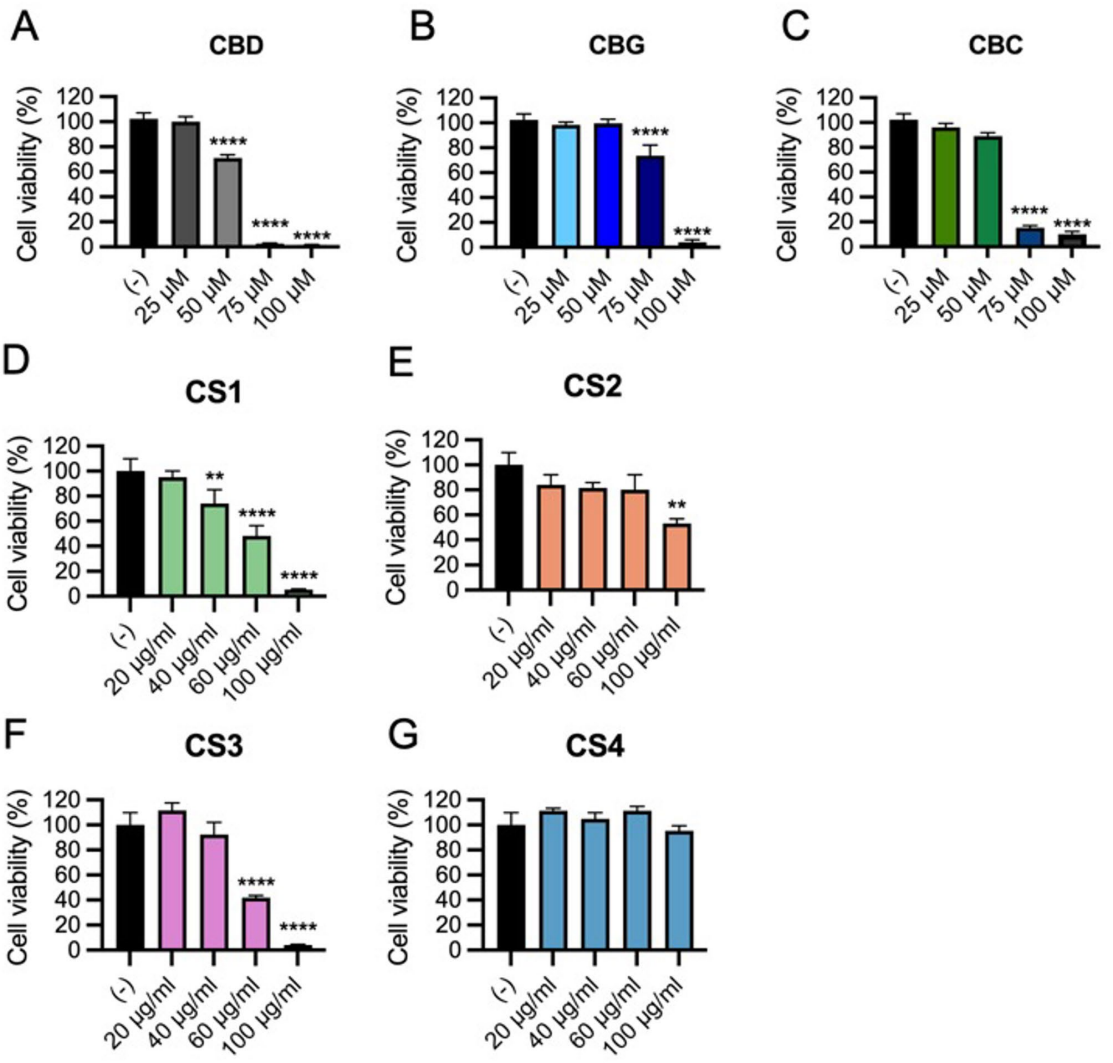
Dose-dependent effect of pure cannabinoids and CS extracts on viability of RAW 264.7 macrophages. Cell viability was evaluated by Alamarblue assay after a-4-hour treatment with CBD (25–100 µM) (**A**), CBG (25–100 µM) (**B**), CBC (25–100 µM) (**C**), (CS1 (20–100 µg/mL) (**D**), CS2 (20–100 µg/mL) (**E**), CS3 (20–100 µg/mL) (**F**), or CS4 (20–100 µg/mL) (**G**). Data are expressed as percentage of cell viability compared to control (vehicle) and shown as mean ± SD of a triplicate. (**p < 0.05, **** *p* < 0.01 vs. CTR; one-way ANOVA)

The dose-dependent effects of CS extracts on viability of RAW 264.7 cells, a murine macrophage cell line that is widely used for evaluating the pro- or anti- inflammatory activities of compounds (Silva et al. 2019) was evaluated. Cells were treated with increasing concentration of extracts (20–100 µg/mL), and pure cannabinoids (25–100 µM) or vehicle for 4 h, followed by viability assessment using the AlamarBlue assay. Results showed that CBD had a significant cytotoxic effect at concentration ≥ 50 µM (Silva et al. 2019), but no viability reduction at 25 µM (Fig. 4A). Both CBG (Fig. 4B) and CBC (Fig. 4C) demonstrated higher biocompatibility than CBD, as they did not affect cell viability up to 50 µM. The CS1 extract, which is the most enriched in CBD (36%), was also the most cytotoxic, with dose-dependent reduction in viability starting at 40 µg/mL (Fig. 4D). In contrast, CS2 and CS3 extracts, respectively CBG and CBC dominant, showed a significant reduction of cell viability only at higher doses, which are 100 µg/mL for CS2 (Fig. 4E) and 60 µg/mL for CS3 (Fig. 4F). The CS4 extract did not impact cell viability even at concentration of 100 µg/mL (Fig. 4G), demonstrating superior biocompatibility. Overall, these results define the maximum non-cytotoxic doses for each extract and pure cannabinoids in RAW 264.7 macrophages, highlighting the role of chemotype-specific composition in cellular responses.

### Evaluation of the anti-inflammatory effects on LPS-induced murine macrophages

To assess the anti-inflammatory effects, RAW-264.7 macrophages were pre-treated with the maximum non-cytotoxic dose of each extract for 30 min, followed by activation with 100 ng/mL of LPS for 4 h. Cells were either untreated (control), treated with LPS alone to induce pro-inflammatory activation, or co-treated with pure cannabinoids as a positive control for anti-inflammatory activity (Kim et al. 2023; Martini et al. 2023). Additional controls included cells treated with CS extracts alone or pure cannabinoids alone.

**Fig. 5:**
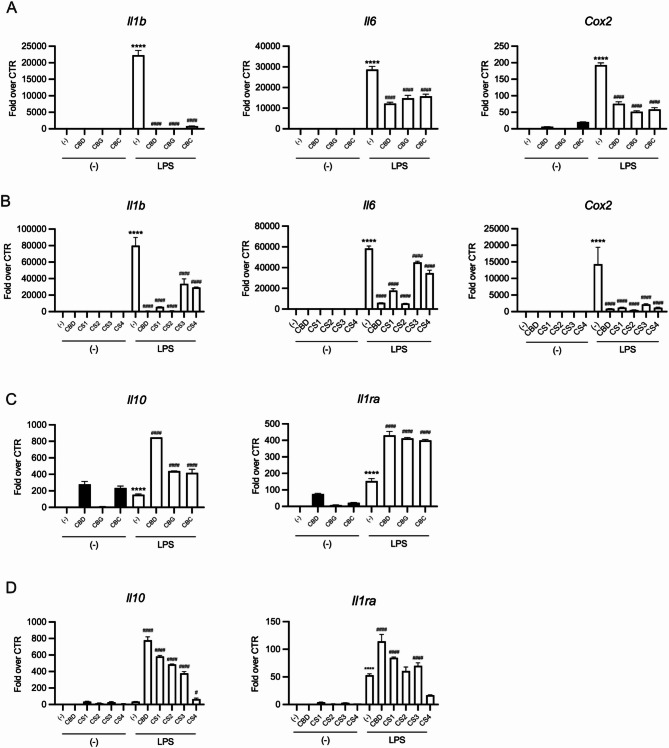
Cannabinoid-containing CS extracts and pure cannabinoids show enhanced anti-inflammatory activity compared to cannabinoid-free extracts. RAW 264.7 cells were pretreated with (**A**, **C**) pure cannabinoids (25 µM CBD, 50 µM CBG, 50 µM CBC) or (B, D) CS extracts (20 µg/mL CS1, 60 µg/mL CS2, 40 µg/mL CS3 and 100 µg/mL CS4) for 30 min, followed by a 4-hour treatment with 100ng/mL of LPS. mRNA levels of (**A**, **B**) pro-inflammatory (I1lb, Il6, Cox2) and (**C**, **D**) anti-inflammatory (Il10, Il1ra) were evaluated by qPCR. *Gapdh* was used as housekeeping gene. Normalized data are shown as fold increase over untreated cells (-). Data are depicted as mean ± SD and are representative of one out of three independent experiments with similar results. *p < 0.05; **p < 0.01; ***p < 0.001; ****p < 0.0001 vs. untreated cells (-); #*p* < 0.05 ####*p* < 0.0001 vs. LPS-induced cells

The expression of key inflammatory (*Il1b*,* Il6*,* Cox2*) and anti-inflammatory (*Il10*,* IL1Ra*) genes using qPCR was evaluated. As expected, LPS significantly upregulated *Il1b*, *Il6*, and *Cox2* (Fig. 5A, B) transcripts, which were markedly reduced by 25µM CBD, 50 µM CBG, or 50 µM CBC co-treatment.

Similarly, 20 µg/mL of CS1, 60 µg/mL of CS2, 40 µg/mL of CS3, containing approximately the same quantity of the relative predominant cannabinoid used as pure compound, significantly inhibited LPS-induced inflammatory gene expression, supporting the pivotal role of cannabinoids in the biological activity (Fig. 5B). Notably, the cannabinoid-free CS4 extract also inhibited LPS-induced inflammatory gene expression (Fig. 5B), underscoring a contribution of non-cannabinoid phenolic compounds to the biological activity of CS extracts. Neither pure cannabinoids nor CS extracts alone significantly affected gene expression in untreated cells. Moreover, pure cannabinoids and cannabinoid-containing extracts (CS1, CS2, CS3) notably upregulated *Il10* and *Il1ra* gene expression, particularly in combination with LPS (Fig. 5C, D). In contrast, the CS4 showed minimal induction of these anti-inflammatory cytokine genes, whether used alone or in combination with LPS (Fig. 5D).

Overall, these findings indicate that both cannabinoid-containing and cannabinoid-free CS extracts effectively reduce the expression of genes encoding for key inflammatory cytokines, such as IL-1β and IL-6, as well as COX2, a crucial enzyme in the synthesis of inflammatory lipids. Furthermore, the selective induction of IL-10 and ILRa by pure cannabinoids and cannabinoid-containing extracts suggests an additional cannabinoid-dependent mechanism that enhances the anti-inflammatory properties of CS1, CS2, and CS3 extracts compared to CS4.

## Discussion

The phytochemical investigation of the non-psychotropic strains of cannabis stated the classification in chemotypes including the new CS3 sample where CBC is the major cannabinoid. Regarding this latter chemotype, the presence of the racemate cannabicitran (CBTC) could be explained by its biosynthetic pathway from CBC or either from Δ^9^-*cis*-THC, which is known to co-occur as a scalemic mixture with Δ^9^-*trans*-THC in cannabis fibre CS strains (Wood et al. 2023). Concerning the non-cannabinoid phenolic compounds, canniprene is not only the most relevant in cannabis for the biological activity, but also the major dihydrostilbenoid with a maximum concentration reported of 0.076% (Allegrone et al. 2017). Following the results, its concentration in CS3 (0.417%) is far superior to that reported by Allegrone and colleagues and still showed the inverse relationship with the concentration of cannflavin A and B (Allegrone et al. 2017; O’Croinin et al. 2023). Moreover, CS3 is the strain that contains the highest concentration ever found of the early discovered 5-methoxy-dihydrodenbinobin (1.12%) firstly identified in chemotype V by Salamone and colleagues in traces (0.0004%) (Salamone et al. 2022).

Regarding the investigation of the antioxidant potential, the lower activity of CS4 suggests that in the extracts, cannabinoids are the principal molecules responsible for neutralizing the DPPH radical and the ABTS assay, aligning with the DPPH assay findings. However, the discrepancy with the DPPH assay related to the pure cannabinoids results may be attributed to the different mechanisms of the experiments: access to the active center of the DPPH radical is more difficult than for the ABTS cation radical. Moreover, the FRAP assays, which measure the reduction of ferric iron (Fe^3+^) to ferrous iron (Fe^2+^), reveal that CS3 may contain non-cannabinoid compounds with electron-donating capabilities. This could be attributed to the content of 5-methoxy-dihydrodenbinobin effective at reducing ferric ions (unpublished data). These findings are consistent with, who emphasized that cannabinoids exhibit different antioxidant mechanisms, and their activity can vary depending on the assay.

Similarly to previous studies (Aiemsaard et al. 2022), our findings confirm that the antibacterial effects of CS extracts are mainly confined to Gram-positive bacteria, with *B. cereus* and *S. epidermidis* the most susceptible. Comparing the extracts, CS1 showed the most potent activity, results supported by the tests performed on pure cannabinoids, where CBD exhibited the strongest inhibitory effects, followed by CBG and CBC, being this latter cannabinoid particularly active against *S. epidermidis*. The cannabinoid-free extract CS4 showed no antibacterial activity across all tested strains, underlining the central role of cannabinoids in mediating the observed effects and supporting the value of chemotype V as a possible negative control in future studies. This interpretation is further strengthened by the adjusted MIC values, recalculated based on the predominant cannabinoid concentration within each extract. These normalized MICs closely matched those of the pure cannabinoids, reinforcing the conclusion that the antimicrobial activity is largely attributable to cannabinoid content rather than to other co-extracted compounds. On the other hand, none of the CS extracts were active against Gram-negative bacteria, probably due to the structural barrier created by the LPS-containing outer membrane, which limits the penetration of hydrophobic molecules like cannabinoids (Denyer and Maillard 2002). This selectivity towards Gram-positive strains, while restraining broader antimicrobial applications, could be advantageous for targeted therapies, such as in dermal or topical formulations where Gram-positive bacteria are predominant.

The MBC assays allowed further refinement of the interpretation of antimicrobial efficacy. Regarding *S. aureus*, MRSA, and *B. cereus*, MBC values were either identical or similar to MIC values, thus indicating a strong killing effect. On the other hand, the slightly higher MBCs observed for *S. epidermidis* and *L. monocytogenes*, suggested a more limited bactericidal activity, potentially reflecting differences in cell wall structure or resistance mechanisms. These findings were supported by the time–kill assay, where all the three cannabinoid-rich extracts reduced the MRSA population by ≥ 3-log CFU/mL. Interestingly, CS2 and CS3 reduced the MRSA counts in just 2 h, demonstrating a rapid and constant bactericidal effect, whereas CS1 achieved the same level in 18 h, suggesting a more gradual but still effective antibacterial action.

Together, these results support the hypothesis that specific chemotypes influence the strength and timing of antimicrobial activity as exemplified by the superior performance of CS2 and CS3, particularly in the time–kill assay that warrants further investigation.

It is well known that the endocannabinoid system (ECS) modulates immune responses by regulating inflammation through the cannabinoid receptors (CB1/CB2) (Rakotoarivelo et al. 2024). Particularly, activation of CB2, which is primarily expressed by immune cells, suppresses pro-inflammatory cytokines (e.g. IL-1β, IL-6, TNF-α) while promoting anti-inflammatory mediators (e.g. IL-10, TGF-β). Cannabinoids mimic these effects, reducing inflammation via receptor-dependent and independent pathways (e.g., PPARγ, TRPV) (Iannotti and Vitale 2021). Noteworthy, despite differences in viability effects, which are likely due to the presence of cannabinoids, all extracts, including the cannabinoid-free CS4, effectively suppressed LPS-induced inflammatory genes (IL-1β, IL-6, Cox2), suggesting that non-cannabinoids phenolic compounds could play a part in the broad anti-inflammatory properties. However, only cannabinoid-containing extracts (CS1, CS2 and CS3) significantly upregulated the anti-inflammatory cytokine IL-10 and IL-1Ra, indicating an additional immunomodulatory mechanism dependent on cannabinoids. These findings highlight that while non-cannabinoid metabolites contribute to anti-inflammatory activity, cannabinoids play a key role in enhancing anti-inflammatory responses via IL-10 and IL-1Ra induction, supporting their therapeutic potential in inflammatory conditions.

## Conclusions

In this study, we provided a phytochemical characterization and biological activity of non-psychoactive *Cannabis sativa* L. extracts from III, IV, V and the emerging CBC chemotype. The phytochemical profile confirmed the distinct percentage of cannabinoid and non-cannabinoid composition of each chemotype, with the CS3 sample exhibiting the highest levels of canniprene and 5-methoxy-dihydrodenbinobin. Antioxidant assays demonstrated that cannabinoids significantly contribute to the radical scavenging capacity of the extracts, with an additional support from non-cannabinoid phenolics as testified by the CS4. Antimicrobial assays showed that only the cannabinoid-containing extracts exhibited potent bactericidal activity against Gram-positive pathogens, including drug-resistant MRSA, while the cannabinoid-free extract lacked such activity. Furthermore, all extracts, including the cannabinoid-free one, were able to suppress LPS-induced pro-inflammatory gene expression in macrophages. However, only the cannabinoid-rich extracts promoted the anti-inflammatory cytokines IL-10 and IL-1Ra, underscoring a cannabinoid-dependent immunomodulatory effect. Taken together, these results highlight the importance of cannabinoid in the biological properties of *Cannabis sativa with a contribution apported by non-cannabinoid phenolic compounds*. Moreover, the anti-inflammatory, antimicrobial, and antioxidant effects observed with both pure cannabinoids and cannabinoid-containing extracts support their potential use in topical formulation for the treatment of chronic inflammatory skin disorders, such as atopic dermatitis and psoriasis. These conditions are often exacerbated by skin dysbiosis and colonization by Gram-positive bacteria like *Staphylococcus aureus*, which contribute to skin barrier dysfunction and amplify immune dysregulation (Zhang et al. 2025). Therefore, while the cannabinoid-free chemotype V serves as a valuable control for dissecting the specific contributions of individual cannabinoids within CS extracts, our findings pave the way for future investigations into the therapeutic potential of selected cannabis-derived products—particularly in the context of antimicrobial resistance and inflammatory diseases associated with dysbiosis.

## Supplementary Information

Below is the link to the electronic supplementary material.

Supplementary Material 1

Supplementary Material 2

## Data Availability

The authors declare that the data supporting the findings of this study are available within the paper and its Supplementary Information files. Should any raw data files be needed in another format they are available from the corresponding author upon reasonable request.

## Abbreviations

ABTS– 2: 2′-Azino-bis(3-ethylbenzothiazoline-6-sulfonic acid)
CBC: Cannabichromene
CB1: Cannabinoid receptor Type 1
CB2: Cannabinoid receptor Type 2
CBD: Cannabidiol
CBG: Cannabigerol
CBTC: Cannabinoid-based topical cream (context-dependent; confirm specific meaning)
CFU: Colony-forming units
COX2: / Cox2 cyclooxygenase-2
DPPH– 2: 2-Diphenyl-1-picrylhydrazyl (radical scavenging assay)
Δ9-THC/ ∆9-THC: Delta-9-tetrahydrocannabinol
∆9-THCA: Delta-9-tetrahydrocannabinolic acid
ECS: Endocannabinoid system
FRAP: Ferric reducing antioxidant power
IL-1β/ *Il1b*: Interleukin-1 beta
IL-6/ *Il6*: Interleukin-6
IL-10/ *Il10*: Interleukin-10
IL1Ra/ *IL1Ra*: Interleukin-1 receptor antagonist
LPS: Lipopolysaccharide
MBC: Minimum bactericidal concentration
mPGES: Microsomal prostaglandin e synthase
mRNA: Messenger ribonucleic acid
MRSA: Methicillin-resistant staphylococcus aureus
MIC: Minimum inhibitory concentration
PPARγ: Peroxisome proliferator-activated receptor gamma
qPCR: Quantitative polymerase chain reaction
TE: Trolox equivalents
TGF-β: Transforming growth factor beta
TNF-α: Tumor necrosis factor alpha
TRPV: Transient receptor potential vanilloid
UHPLC-HRMS: Ultra-high-performance liquid chromatography–high-resolution mass spectrometry
