# Supplementary material for: Targeting the antioxidant, antimicrobial and anti-inflammatory activity of non-psychotropic Cannabis sativa L.: a comparison with chemotype V
Source: J Cannabis Res. 2025 Oct 21;7:79. doi: 10.1186/s42238-025-00336-1 (PMC12542492; doi:10.1186/s42238-025-00336-1)
Supplement: Supplementary file 1 — Supplementary Material 1 [file 42238_2025_336_MOESM1_ESM.docx]

**Targeting the antioxidant, antimicrobial and anti-inflammatory activity of non-psychotropic *Cannabis sativa* L.: a comparison with chemotype V**

Chiara Ceresa^1^ ^†^, Martina Delsignore^1,3†^, Matej Maly^2^, Francesca Carrà^1^, František Beneš^2^, Andrea Chiara Sansotera^1^, Aurora Camola^1^, Marco Arlorio^1^, Chiara Porta^1,3^, Letizia Fracchia^1^, Vincenzo Disca^1*^ and Federica Pollastro^1*^

^1^University of Piemonte Orientale, Department of Pharmaceutical Sciences, L.go Donegani 2, 28100 Novara, Italy.

^2^University of Chemistry and Technology in Prague, Department of Food Analysis and Nutrition, Technická 5, 166 28 Prague 6, Czech Republic

^3^University of Piemonte Orientale, Center for Translational Research on Autoimmune & Allergic Diseases (CAAD), C.so Trieste 15/A, 28100 Novara, Italy.

1. **Materials and methods:**

**1.1 General experimental procedures**

**1.2 Bacterial strains and reagents**

**1.3 Cell cultures and reagents**

**1.4 Plant material**

**1.5 Extraction of cannabis inflorescences**

**1.6 Cannabinoids isolation**

**1.7 Antioxidant activity assays**

**1.8 Quantitative analysis of cannabinoids and non-cannabinoid phenolic compounds with UHPLC-HRMS**

**1.9 MICs determination**

**1.10 MBCs determination**

**1.11 Time-kill assay**

**1.12 Cell viability**

**1.13 Cell treatments**

**1.14 Gene expression analysis**

1. **References**

Table S1. Primer sequences used for qPCR

Table S2. Cannabinoids content expressed as mg/Kg of extracts

Table S3. Flavonoids content expressed as mg/Kg of extracts

Fig. S1: ^1^H NMR (400 MHz) of cannabidiol (CBD 1) in CDCl3

Fig. S2: ^1^H NMR (400 MHz) of cannabigerol (CBG 2) in CDCl3

Fig. S3: ^1^H NMR (400 MHz) of cannabichromene (CBC 3) in CDCl3

**1.1 General experimental procedures**

Silica gel 60 (0.063−0.200 mm) and Celite® 545 (0.02−0.1 mm), used for low-pressure liquid chromatography (LPC), were purchased from Macherey-Nagel (Düren, Germany). Purifications were monitored by TLC 60 F254 (0.25 mm) plates purchased from Merck (Darmstadt, Germany) and visualized by staining with 5% H_2_SO_4_ in EtOH and heating. Organic solvents and reagents were supplied by Sigma-Aldrich (Milan, Italy). Anhydrous ethanol was supplied by Carlo-Erba (Milan, Italy). ^1^H (400 MHz) spectra were measured on Bruker 400 spectrometers (Bruker, Billerica, MA, USA). A HPLC JASCO Hichrom silica (250 × 25 mm), UV-vis detector-2075 plus (Oklahoma, Japan) was used. Chemical shifts were referenced to the residual solvent signal (CDCl_3_: *δ*_H_ = 7.26). Spectrophotometric analyses were carried out on a Shimadzu UV-1900 272 spectrophotometer (Shimadzu, Tokyo, Japan). A Q-Exactive Plus UHMR Hybrid Quadrupole Orbitrap™ Mass Spectrometer equipped with a Vanquish™ Duo UHPLC system (Waltham, MA, USA) was used for quantification of cannabinoids and flavonoids. Xcalibur™ 4.0 (Thermo Scientific, San José, California) was used for the calculation of the exact mass of the analytes and data processing. Statistical analysis for differences in antioxidant activity and the content of cannabinoids and non-cannabinoids compounds in the various extracts was performed using the software R4.3.1 (Boston, USA). All the results were expressed as mean ± standard deviation (SD) of three independent experiments. Differences were estimated by analysis of variance (ANOVA) followed by Tukey’s honest significant test and the statistical significance level was set at 0.05.

**1.2 Bacterial strains and reagents**

*Staphylococcus aureus* ATCC 6538, methicillin-resistant *Staphylococcus aureus* (MRSA) ATCC 43300, *Staphylococcus epidermidis* ATCC 12228, *Bacillus cereus* ATCC 10876, *Listeria monocytogenes* ATCC 19115, *Escherichia coli* ATCC 25922 and *Salmonella enterica* ATCC 13311 were obtained from the American Type Culture Collection (ATCC - Manassas, VA, USA). Bacteria were cultured in Mueller-Hinton Broth (MHB), Tryptic Soy Broth (TSB), Mueller-Hinton Agar (MHA) and Tryptic Soy Agar (TSA) supplied by Scharlab (Barcelona, Spain).

**1.3 Cell cultures and reagents**

Murine RAW 264.7 macrophage cells were maintained in Dulbecco’s Modified Eagle’s Medium with High Glucose (DMEM; EuroClone S.p.A., Assago, MI, Italy), supplemented with 10% Fetal Bovine Serum (FBS; EuroClone), 100 U/mL penicillin, 100 µg/mL streptomycin (both from EuroClone), and 2 mM L-glutamine (EuroClone). Cells were cultured at 37 °C in a humidified incubator with 5% CO_2_. Mycoplasma contamination was routinely checked using the MycoBlue Mycoplasma Detector (Vazyme Biotech Co., Ltd., Nanjing, China).

AlamarBlue™ reagent was purchased from Bio-Rad Laboratories, Inc. (Hercules, CA, USA). Fluorescence measurements were performed using a TECAN SPARK spectrophotometer (TECAN Group Ltd., Männedorf, Switzerland). Dimethyl sulfoxide (DMSO) was purchased from Carlo Erba Reagents S.A.S. (Milan, Italy). Lipopolysaccharide (LPS) from *Salmonella abortus equi* S-form (TLRGRADE®) was purchased from Enzo Life Sciences (Catalog number ALX-581-009, Farmingdale, NY, USA). RNA was extracted using PUREzol reagent (Bio-Rad Laboratories, Inc., Hercules, CA, USA), and RNA purity was assessed using a NanoDrop spectrophotometer (Thermo Fisher Scientific, Madison, WI, USA). The High-Capacity cDNA Reverse Transcription Kit was purchased from Thermo Fisher Scientific. SsoAdvanced Universal SYBR Green Supermix and the CFX96 thermocycler were purchased from Bio-Rad Laboratories, Inc. Primer sets for all amplicons were designed using the IDT Primer Quest Tool (Integrated DNA Technologies, IDTNA).

**1.4 Plant material**

*Cannabis sativa* inflorescences have been supplied by Canvasalus S.r.l. (Monselice, PD, Italy): *C. sativa* III chemotype CBD rich (Cs-CBD05/2022 and Cs-Carmagnola20), *C. sativa* IV chemotype CBG rich (Cs-CBG12/2022), *C. sativa* chemotype CBC rich (Cs-CBC01/2023), *C. sativa* V chemotype (Cs-nocann02/2022). Vouchers specimens of the vegetable material are stored in Novara Laboratories.

**1.5 Extraction of cannabis inflorescences**

Ethanolic extracts were obtained from 100 g of each chemotype inflorescences (homogenized hemp). The samples were extracted with ethanol (inflorescences/ethanol ratio 1:5 w/v, 2 x 12 h) in a macerator at room temperature. After the extraction, the vegetal material was removed by filtration using Whatman No 42-filter paper. The solvent was evaporated at reduced pressure to afford each extract as black syrups (Table 1).

The extraction yield was expressed in % as:

(g of ethanolic extract) / (g of homogenized hemp) * 100

**1.6 Cannabinoids isolation**

*C. sativa* inflorescences (57 g) belonging to chemotype III (voucher specimen Cs-Carmagnola20) were extracted with acetone (inflorescences/acetone ratio 1:10 w/v) in a vertical percolator at room temperature, affording 4 g (7%) of a dark green syrup after evaporation of the solvent at reduced pressure. This was later dissolved at 45 °C in 30 mL of MeOH (with a raw extract/MeOH ratio corresponding to 1:10 w/v) and left at 8 °C to condense fatty acids and waxes. After 12 h, the solution was vacuum filtered with cold MeOH (50 mL) in a sintered funnel protected by a bed of stratified Celite*®*, obtaining 2.9 g of the residual methanolic fraction. This latter portion was filtered through solid-phase extraction on C-18 silica gel (30 g) to remove pigments and unsaturated fatty acids. For this purpose, the fraction was charged on 25 g C-18 silica gel (with a raw extract/stationary phase ratio of 1:10 w/w), packed with MeOH in a sintered funnel (4 × 10 cm) with a side arm for vacuum. Elution with MeOH (100 mL) gave 2.4 g of the purified fraction after evaporation of the solvent at reduced pressure. Once the fraction was dried, it was heated at 130 °C under stirring for 45 °C in a paraffin bath to achieve the decarboxylation followed by TLC (silica PE/EtOAc 70:30 v/v). This latter decarboxylated fraction was fractionated by LPC on silica gel (50 g, PE–EtOAc gradient from 90:10 to 20:80 v/v) to afford three fractions (I, II, and III). Fraction I (745 mg) was further purified with HPLC (250 × 25 mm silica, PE–EtOAc gradient from 90:10 to 80:20 v/v) to afford 180 mg of CBD 1 (Choi et al., 2004) as a white powder, 35 mg of CBC 3 (Claussen et al., 1966) as brownish powder, and 88 mg of CBG 2 (Choi et al., 2004) as a white powder. All the isolated compounds were identified according to ^1^H NMR previously described in the literature. NMR data of the isolated compounds are shown in Fig. S1-S3.

**1.7 Antioxidant activity assays**

The assessment of radical scavenging activity was assessed by different assay: the inhibition of the DPPH^•^ and ABTS^•+^ radicals, and by the Ferric ion antioxidant power (FRAP) following the methodology reported elsewhere (Disca et al., 2024; Jaouhari et al., 2024). Briefly, the extracts and the pure compounds were solubilized in ethanol and were subjected to triplicate assays. The outcomes were expressed as g of Trolox equivalent (TE) per kg of extract/compound.

**1.8 Quantitative analysis of cannabinoids and non-cannabinoid phenolic compounds with UHPLC-HRMS**

Phytochemical investigation of samples occurred by UHPLC-HRMS on RP C-18 following the method described by Benes et al., (2024) (solvent A: water-MeOH 95:5, v/v with 5 mM ammonium formate and 0.1% formic acid, solvent B: isopropyl alcohol-MeOH-water 65:30:5, v/v/v with 5 mM ammonium formate and 0.1% formic acid). The total run time was 16 minutes with a 3 μL injection volume and gradient elution: 5% B, increase to 60% B by 1 minute, increase to 70% B by 11 minutes, rapid increase to 100% B in 0.5 minutes, isocratic elution for 2 minutes and return to initial conditions for 2.5 minutes. Cannabinoids and non-cannabinoid phenolic compounds were identified by orbital trap mass spectrometer with the following ESI± parameters: sheath/aux gas (N2) flow of 45/10 arb. U., aux gas temperature of 300 °C, spray voltage of 3.5 kV and S-lens RF level of 55. The mass spectrometer operated in Full scan mode: 70,000 FWHM resolution, 100–1,000 m/z scan range, AGC target 2e5, maxIT 50 ms. Quantification with Xcalibur™ 4.0 was done using solvent calibration standards (EtOH) containing 39 cannabinoids, 12 flavonoids, the dihydrostilbenoid canniprene and the dihydrophenanthrene 5-methoxy-dihydrodenbinobin. The limits of quantification (LOQs), representing the lowest points of calibration, were 0.50–1 mg/kg. The calibration curves were linear up to 50 mg/kg (R² ≥ 0.999). The measurement uncertainty, expressed as relative standard deviation (RSD), was 6–12%.

**1.9 MICs determination**

The MICs for hemp extracts, pure cannabinoids and control antibiotics (tetracycline, ciprofloxacin, linezolid, methicillin) were evaluated by the broth microdilution method described by Wiegand et al., (2008) with minor changes. Bacteria (~5 × 10^5^ Colony Forming Unit per mL—CFU/mL) were cultured in MHB (*S. aureus*, MRSA, *S. epidermidis*, *B. cereus*, *E. coli* and *S. enterica*) or TSB (*L. monocytogenes*) in the presence of varying concentrations of hemp extracts and pure compounds (test wells), control antibiotics, or 0.5% v/v DMSO (used as a positive control for growth) and incubated for 16–20 h at 37 °C.

The MIC was defined as the lowest concentration of the compound that visibly inhibited bacterial growth. The assays were performed in triplicate and repeated in three independent experiments (n = 9).

**1.10 MBCs determination**

The MBCs of hemp extracts was determined as described by Mohammad et al., (2017)*.*  Aliquots of 20 µL were transferred from the wells showing no visible growth to MHA or TSA plates and incubated at 37 °C for 24 h.

The MBC was defined as the lowest concentration of the compound that killed 99.9% of the bacterial population. Assays were conducted in duplicate and repeated in two independent experiments (n = 4).

**1.11 Time-kill assay**

The time-kill assay was performed for hemp extracts and control antibiotics against MRSA ATCC 43300, following the method described by Brunelli et al., (2023). Bacterial cells (~2 × 10^6^ CFU/mL) were grown in MHB in the presence of 4 × MIC of hemp extracts (test wells), ciprofloxacin (bactericidal agent), or 0.5% v/v DMSO (positive control for growth) for 24 h at 37 °C. Samples of 20 μL were collected at 0, 2, 4, 6, 8, 18, and 24 h, serially diluted in 0.9% w/v NaCl, plated on MHA, and incubated at 37 °C for 18 h prior to colony counting.

**1.12 Cell viability**

Cell viability was assessed using AlamarBlue™ reagent (Bio-Rad), according to manufacturer's instructions. Briefly, cells were seeded at 1 × 10⁵ cells/well in 96-well plates and incubated overnight. Cells were treated with increasing concentrations of hemp extracts (20-100 µg/mL) or pure CBD (25-100 µM). Following 4h treatment, culture medium was replaced with fresh medium containing 10% (v/v) AlamarBlue™ reagent. After 3 h of incubation at 37 °C (protected from light), fluorescence was measured using a SPARK TECAN microplate reader (TECAN) with an excitation wavelength of 530–560 nm and an emission wavelength of 590 nm. All assays were performed in triplicate and repeated in three independent biological experiments. Cell viability was calculated in comparison to vehicle (DMSO)-treated controls.

**1.13 Cell treatments**

RAW 264.7 cells were seeded at 4 × 10⁶ cells/well in 6-well plates and incubated overnight. Cells were pre-treated for 30 minutes with the highest non-cytotoxic concentration of hemp extracts (20 µg/mL for CS1, 60 µg/mL for CS2, 40 µg/ml for CS3 and 100 µg/mL for CS4) or vehicle (0,5% v/v). After pre-treatment, cells were stimulated with 100 ng/mL LPS (Salmonella abortus equi S-form; Enzo Life Sciences) for 4 h. Untreated cells were used as control. Cells were lysed with PUREzol reagent (Bio-Rad) for downstream RNA extraction and gene expression analysis.

**1.14 Gene expression analysis**

Total RNA was isolated using PUREzol reagent (Bio-Rad) following the manufacturer’s protocol. Briefly, cells were homogenized in PUREzol, and RNA was phase-separated with chloroform, precipitated with isopropanol, and washed twice with 75% ethanol. The RNA pellet was air-dried and resuspended in RNase-free water. RNA concentration and purity was assessed using a Nanodrop spectrophotometer (Thermo Fisher Scientific). Only samples with a 260/280 and a 260/230 OD ratios > 1.5 were processed further. 2 μg of total RNA was reverse-transcribed using the High-Capacity cDNA Reverse Transcription Kit (Thermo Fisher Scientific) according to the manufacturer’s instructions. Quantitative PCR (qPCR) was performed in triplicate using SsoAdvanced Universal SYBR Green Supermix (Bio-Rad) on a CFX96 thermocycler (Bio-Rad). Gene-specific primers (Table S1) were used for amplification. Inflammatory gene expression levels were normalized to GAPDH as the housekeeping gene and calculated using the 2^(−ΔΔCt) method**.** Results are shown as fold change relative to untreated cells (controls)**.**

Table S1. Primer sequences used for qPCR

| Gene | Forward | Reverse |
| --- | --- | --- |
| *Gapdh* | TTCAACGGCACAGTCAAG | CCAGTAGACTCCACGACATA |
| *Il1b* | AAGTTGACGGACCCCAAAAGAT | TGTTGATGTGCTGCTGCGA |
| *Il6* | GGATACCACTCCCAACAGACCT | GCCATTGCACAACTCTTTTCTC |
| *Cox2*  *Il10*  *Il1ra* | AATGAGTACCGCAAACGCTTC  GCAGGACTTTAAGGGTTACTTGG  TGGCCTAATCCCCATGATGA | CAGCCATTTCCTTCTCTCCTGTA  GGGGCATCACTTCTACCAGG  AGACTTCACCCAGATGGCAGAG |

Table S2. Cannabinoids content expressed as mg/Kg of extracts.

| Compound (mg/kg) | CS1 | CS2 | CS3 | CS4 |  |
| --- | --- | --- | --- | --- | --- |
| CBGO | 5.45 ± 0.65 | 5.78 ± 0.52 | <LOQ | <LOQ |  |
| CBGOA | 4.94 ± 0.27 | 13.59 ± 0.05 | 5.22 ± 0.15 | <LOQ |  |
| CBDVA | 430.16 ± 11.05 | 21.61 ± 0.84 | 30.56 ± 4.51 | 7.72 ± 0.29 |  |
| CBND | 570.42 ± 35.63 | <LOQ | <LOQ | <LOQ |  |
| CBDV | 3662.9 ± 325.29 | 17.5 ± 1.98 | 10.25 ± 0.17 | 8.3 ± 1.21 |  |
| CBGV | 39.31 ± 3.27 | 214.21 ± 16.13 | 42.41 ± 0.26 | <LOQ |  |
| CBGVA | <LOQ | 90.66 ± 1.01 | 129.92 ± 5.54 | <LOQ |  |
| CBDB | 826.26 ± 32.94 | <LOQ | <LOQ | <LOQ |  |
| CBGB | <LOQ | 150.44 ± 11.09 | 58.22 ± 7.63 | <LOQ |  |
| CBE | 4043.57 ± 56.14 | <LOQ | <LOQ | <LOQ |  |
| CBCO | 52.41 ± 0.41 | 70.58 ± 4.49 | 45.89 ± 1.42 | <LOQ |  |
| CBV | 67.59 ± 4 | 41.3 ± 0.29 | 14.75 ± 0.83 | <LOQ |  |
| CBDA | 37962.57 ± 3056.04 | 277.48 ± 34.36 | 7651.57 ± 91.19 | 538.68 ± 93.88 |  |
| CBG | 5214.83 ± 305.09 | 178549.01 ± 7133.88 | 23461.56 ± 301.19 | <LOQ |  |
| CBD | 360585.68 ± 51718.52 | 1041.7 ± 476.7 | 3262.66 ± 455.92 | 45.99 ± 0.97 |  |
| CBVA | 14.02 ± 0.16 | 5.69 ± 0.09 | 4.76 ± 0.44 | <LOQ |  |
| CBGA | 3730.22 ± 9.03 | 89506.85 ± 1455.08 | 37615.99 ± 31.21 | 21.55 ± 1.81 |  |
| THCV | 20.68 ± 1.17 | 20.19 ± 1.49 | 129.94 ± 18.86 | <LOQ |  |
| CBDH | 50.32 ± 0.86 | <LOQ | <LOQ | <LOQ |  |
| CBCV | 209.47 ± 4.77 | 162.85 ± 28.37 | 1831.96 ± 62.49 | <LOQ |  |
| THCVA | <LOQ | <LOQ | 5.13 ± 0.16 | <LOQ |  |
| CBN | 6209.2 ± 396.78 | 1622.12 ± 83.3 | 498.86 ± 20.04 | <LOQ |  |
| CBCVA | 40.38 ± 1.06 | 16.03 ± 0.03 | 196.94 ± 12.67 | <LOQ |  |
| cis-delta-THC | 2684.71 ± 102.8 | 574.42 ± 31.78 | 1313.87 ± 28.59 | <LOQ |  |
| CBDP | 38.1 ± 2.92 | <LOQ | <LOQ | <LOQ |  |
| CBNA | 138.18 ± 1.99 | 35.49 ± 2.05 | 45.91 ± 3.21 | 4.03 ± 0.35 |  |
| THC | 1941.55 ± 66.2 | 880.67 ± 194.45 | 3660.25 ± 619.28 | <LOQ |  |
| CBL | 958.4 ± 79.29 | 682.92 ± 68.76 | 2373.58 ± 30.34 | <LOQ |  |
| CBC | 11326.08 ± 40.21 | 30303.66 ± 1074.21 | 250985.99 ± 17657.37 | 31.71 ± 0.72 |  |
| THCA | 155.47 ± 14.28 | 39.64 ± 0.86 | 255.45 ± 6.21 | 35.87 ± 8.95 |  |
| CBCA | 1426.08 ± 9.51 | 2683.93 ± 43.62 | 29130.89 ± 451.62 | 29.65 ± 1.59 |  |
| CBTC | 5194.69 ± 287.94 | 7312.18 ± 119.54 | 57305.66 ± 1265.81 | 6.49 ± 0.06 |  |
| CBLA | 227.89 ± 15.84 | 195.66 ± 24.98 | 1355.32 ± 136.07 | 5.89 ± 0.38 |  |
| Total | 447831 | 314536 | 421423 | 736 |  |

Different letters indicate statistical differences (p < 0.05), within the same molecule.

Table S3. Flavonoids content expressed as mg/Kg of extracts. Different letters indicate statistical differences (p>0.05) within the same molecule.

| Compound (mg/kg) | CS1 | CS2 | CS3 | CS4 |
| --- | --- | --- | --- | --- |
| Vitexin | < LOQ | < LOQ | < LOQ | 75.05 ± 8.03 |
| Luteolin | 64.36 ± 8.03^c^ | 26.01 ± 8.59^d^ | 231.81 ± 6.3^a^ | 129.53 ± 7.11^b^ |
| Quercetin |  | 15.08 ± 1.87 |  |  |
| Apigenin | 171.23 ± 1.62^c^ | 63.1 ± 8.57^d^ | 462.62 ± 2.05^b^ | 599.98 ± 24.19^a^ |
| Chrysoeriol | 117.5 ± 13.41b^c^ | 8.64 ± 0.47^c^ | 254.39 ± 17.23^b^ | 1711 ± 70.71^a^ |
| Cannflavin B | 1468.34 ± 60.15^b^ | 355.55 ± 10.85^c^ | 2899.43 ± 160.05^a^ | 2965.83 ± 91.02^a^ |
| Cannflavin A | 2557.6 ± 91.14^c^ | 1892.29 ± 66.84^d^ | 7709.13 ± 62.28^b^ | 10802.84 ± 12.55^a^ |
| Morin | 2.96 ± 0.54 | 4.49 ± 0.66 |  | 2.83 ± 0.1 |
| Diosmetin | 93.79 ± 10.73c | 6.56 ± 0.45^d^ | 217.91 ± 1.47^b^ | 826.52 ± 12.81^a^ |
| Baicalein | 18.92 ± 2.55^c^ | 2 ± 0.23^d^ | 62.25 ± 0.7^b^ | 86.22 ± 2.96^a^ |
| Genistein | 11.96 ± 1.35^c^ | 1.29 ± 0.04^d^ | 38.68 ± 1.36^b^ | 51.55 ± 0.97^a^ |
| Canniprene | 502 ± 21^b^ | < LOQ | 4168 ± 337^a^ | 491 ± 16^b^ |
| 5-methoxy-dihydrodenbinobene | < LOQ | < LOQ | 11168 ± 74^a^ | 940 ± 108^b^ |
| Total | 5008^c^ | 2375^d^ | 27212^a^ | 18682^b^ |

Different letters indicate statistical differences (p < 0.05), within the same molecule.


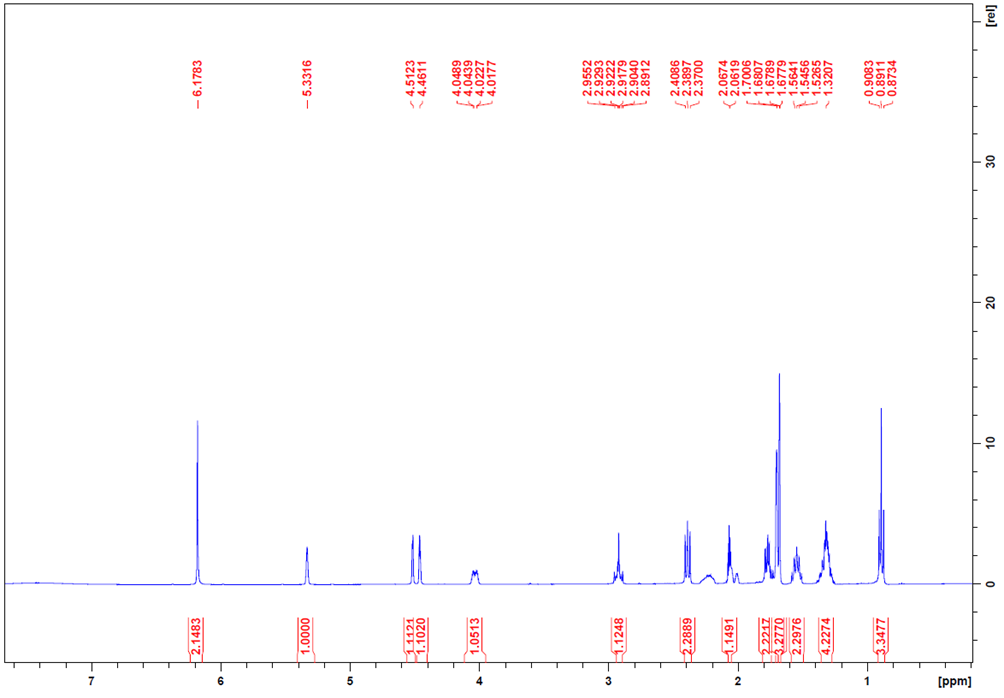


Fig. S1: ^1^H NMR (400 MHz) of cannabidiol (CBD 1) in CDCl_3_


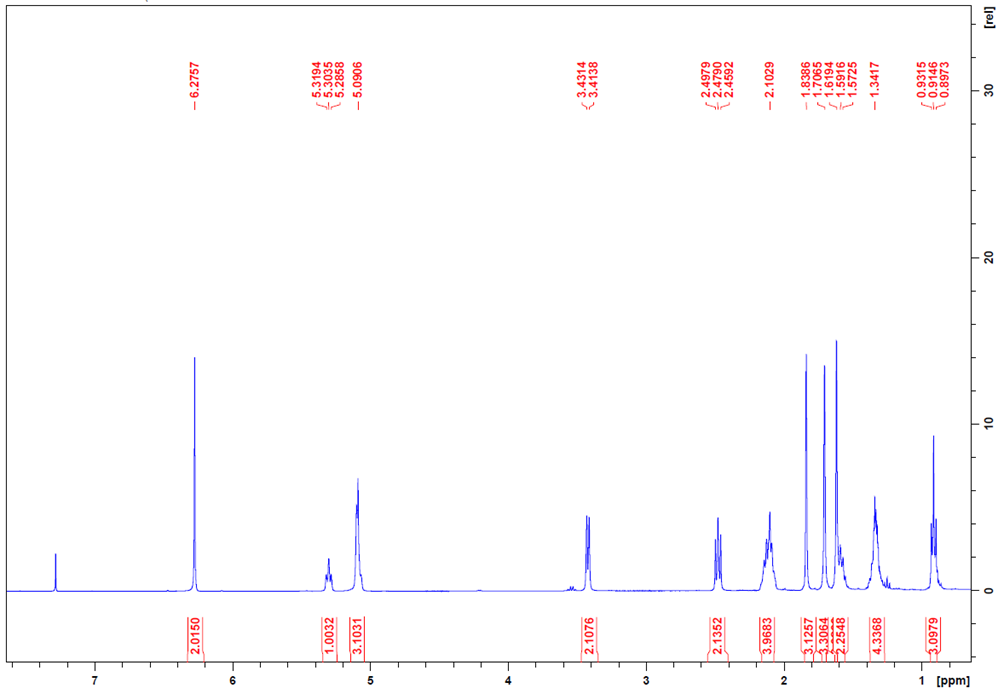
Fig. S2: ^1^H NMR (400 MHz) of cannabigerol (CBG 2) in CDCl_3_


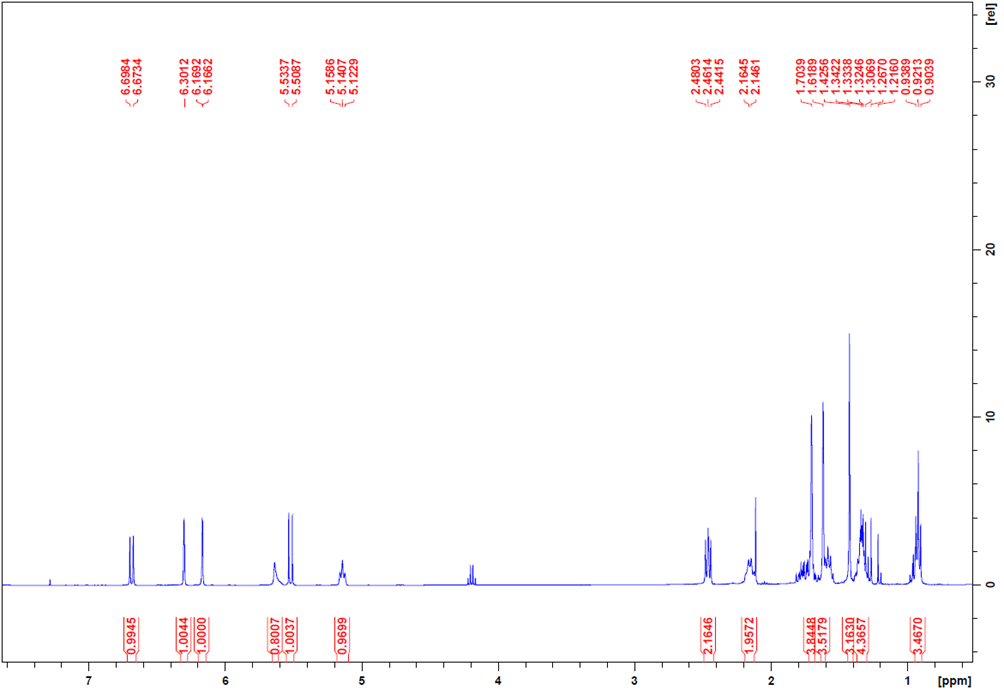


Fig. S3: ^1^H NMR (400 MHz) of cannabichromene (CBC 3) in CDCl_3_

**2. References**

Benes, F., Binova, Z., Zlechovcova, M., Maly, M., Stranska, M., & Hajslova, J. (2024). Thermally induced changes in the profiles of phytocannabinoids and other bioactive compounds in *Cannabis sativa* L. inflorescences. *Food Research International*, *190*, 114487. https://doi.org/10.1016/j.foodres.2024.114487

Brunelli, F., Ceresa, C., Aprile, S., Coppo, L., Castiglioni, B., Bosetti, M., Fracchia, L., & Tron, G. C. (2023). Isocyanides in med chem: A scaffold hopping approach for the identification of novel 4-isocyanophenylamides as potent antibacterial agents against methicillin-resistant *Staphylococcus* *aureus*. *European Journal of Medicinal Chemistry*, *246*, 114950. https://doi.org/10.1016/j.ejmech.2022.114950

Disca, V., Jaouhari, Y., Carrà, F., Martoccia, M., Travaglia, F., Locatelli, M., Bordiga, M., & Arlorio, M. (2024). Effect of Carbohydrase Treatment on the Dietary Fibers and Bioactive Compounds of Cocoa Bean Shells (CBSs). *Foods*, *13*(16), Article 16. https://doi.org/10.3390/foods13162545

Jaouhari, Y., Disca, V., Ferreira-Santos, P., Alvaredo-López-Vizcaíno, A., Travaglia, F., Bordiga, M., & Locatelli, M. (2024). Valorization of Date Fruit (Phoenix dactylifera L.) as a Potential Functional Food and Ingredient: Characterization of Fiber, Oligosaccharides, and Antioxidant Polyphenols. *Molecules*, *29*(19), Article 19. https://doi.org/10.3390/molecules29194606

Mohammad, H., Younis, W., Chen, L., Peters, C. E., Pogliano, J., Pogliano, K., Cooper, B., Zhang, J., Mayhoub, A., Oldfield, E., Cushman, M., & Seleem, M. N. (2017). Phenylthiazole Antibacterial Agents Targeting Cell Wall Synthesis Exhibit Potent Activity in Vitro and in Vivo against Vancomycin-Resistant Enterococci. *Journal of Medicinal Chemistry*, *60*(6), 2425–2438. https://doi.org/10.1021/acs.jmedchem.6b01780

Wiegand, I., Hilpert, K., & Hancock, R. E. W. (2008). Agar and broth dilution methods to determine the minimal inhibitory concentration (MIC) of antimicrobial substances. *Nature Protocols*, *3*(2), 163–175. https://doi.org/10.1038/nprot.2007.521
