# Supplementary material for: Targeting the antioxidant, antimicrobial and anti-inflammatory activity of non-psychotropic Cannabis sativa L.: a comparison with chemotype V
Source: J Cannabis Res. 2025 Oct 21;7:79. doi: 10.1186/s42238-025-00336-1 (PMC12542492; doi:10.1186/s42238-025-00336-1)
Supplement: Supplementary file 2 — Supplementary Material 2 [file 42238_2025_336_MOESM2_ESM.docx]

**Targeting the antioxidant, antimicrobial and anti-inflammatory activity of non-psychotropic *Cannabis sativa* L.: a comparison with chemotype V**

Chiara Ceresa^1^ ^†^, Martina Delsignore^1,3†^, Matej Maly^2^, Francesca Carrà^1^, František Beneš^2^, Andrea Chiara Sansotera^1^, Aurora Camola^1^, Marco Arlorio^1^, Chiara Porta^1,3^, Letizia Fracchia^1^, Vincenzo Disca^1*^ and Federica Pollastro^1*^

^1^University of Piemonte Orientale, Department of Pharmaceutical Sciences, L.go Donegani 2, 28100 Novara, Italy.

^2^University of Chemistry and Technology in Prague, Department of Food Analysis and Nutrition, Technická 5, 166 28 Prague 6, Czech Republic

^3^University of Piemonte Orientale, Center for Translational Research on Autoimmune & Allergic Diseases (CAAD), C.so Trieste 15/A, 28100 Novara, Italy.

***Corresponding authors:** vincenzo.disca@uniupo.it; federica.pollastro@uniupo.it

**List of Abbreviations**

LPC: Low Pressure Chromatography

TLC: Thin Layer Chromatography

HPLC: High-Performance Liquid Chromatography

HRMS: High-Resolution Mass Spectrometry

UHPLC: Ultra-High Performance Liquid Chromatography

MHB: Mueller-Hinton Broth

TSB: Tryptic Soy Broth

MHA: Mueller-Hinton Agar

TSA: Tryptic Soy Agar

DMEM: Dulbecco’s Modified Eagle Medium

FBS: Fetal Bovine Serum

CBD: Cannabidiol

CBG: Cannabigerol

CBC: Cannabichromene

DPPH: 2,2-Diphenyl-1-picrylhydrazyl

ABTS: 2,2'-Azino-bis(3-ethylbenzothiazoline-6-sulfonic acid)

FRAP: Ferric Reducing Antioxidant Power

TE: Trolox Equivalent

ESI: Electrospray Ionization

FWHM: Full Width at Half Maximum

XIC: Extracted Ion Chromatogram

MIC: Minimum Inhibitory Concentration

MRSA: Methicillin-Resistant Staphylococcus aureus

DMSO: Dimethyl Sulfoxide

MBC: Minimum Bactericidal Concentration

CFU: Colony Forming Unit

OD: Optical Density

qPCR: Quantitative Polymerase Chain Reaction

ANOVA: Analysis of Variance

HSD: Honestly Significant Difference

**1. MATERIALS AND METHODS**

**1.1 General experimental procedures**

Silica gel 60 (0.063−0.200 mm) and Celite® 545 (0.02−0.1 mm), used for low-pressure liquid chromatography (LPC), were purchased from Macherey-Nagel (Düren, Germany). Purifications were monitored by TLC 60 F254 (0.25 mm) plates purchased from Merck (Darmstadt, Germany) and visualized by staining with 5% H_2_SO_4_ in EtOH and heating. Organic solvents and reagents were supplied by Sigma-Aldrich (Milan, Italy). Anhydrous ethanol was supplied by Carlo-Erba (Milan, Italy). ^1^H (400 MHz) spectra were measured on Bruker 400 spectrometers (Bruker, Billerica, MA, USA). A HPLC JASCO Hichrom silica (250 × 25 mm), UV-vis detector-2075 plus (Oklahoma, Japan) was used. Chemical shifts were referenced to the residual solvent signal (CDCl_3_: *δ*_H_ = 7.26). Spectrophotometric analyses were carried out on a Shimadzu UV-1900 272 spectrophotometer (Shimadzu, Tokyo, Japan). A Shimadzu UV-1900 272 spectrophotometer (Shimadzu, Tokyo, Japan) was utilized for the antioxidant activity assays. A Q-Exactive Plus HRMS Hybrid Quadrupole Orbitrap™ Mass Spectrometer equipped with a Vanquish™ Duo UHPLC system (Waltham, MA, USA) was used for quantification of cannabinoids and flavonoids. Xcalibur™ 4.0 (Thermo Scientific, San José, California) was employed for the calculation of the exact mass of the analytes and data processing.

**1.2 Bacterial strains and reagents**

*Staphylococcus aureus* ATCC 6538, methicillin-resistant *Staphylococcus aureus* (MRSA) ATCC 43300, *Staphylococcus epidermidis* ATCC 12228, *Bacillus cereus* ATCC 10876, *Listeria monocytogenes* ATCC 19115, *Escherichia coli* ATCC 25922 and *Salmonella enterica* ATCC 13311 were obtained from the American Type Culture Collection (ATCC - Manassas, VA, USA). Bacteria were cultured in Mueller-Hinton Broth (MHB), Tryptic Soy Broth (TSB), Mueller-Hinton Agar (MHA) and Tryptic Soy Agar (TSA) supplied by Scharlab (Barcelona, Spain). Stock solutions of ciprofloxacin (200 µg/mL) (Sigma-Aldrich, Milan, Italy), tetracycline (400 µg/mL) (Sigma-Aldrich, Milan,Italy), linezolid (400 µg/mL) (Tokyo Chemical Industry Co., Ltd.) and methicillin (3.2 mg/mL) (Sigma-Aldrich, Milan, Italy) were prepared in DMSO (Sigma-Aldrich, Milan, Italy), stored at 4 °C, and subsequently diluted in sterile deionized water to the working concentrations before the assays.

**1.3 Cell cultures and reagents**

Murine RAW 264.7 macrophage cells were maintained in Dulbecco’s Modified Eagle’s Medium with High Glucose (DMEM; EuroClone S.p.A., Assago, MI, Italy), supplemented with 10% Fetal Bovine Serum (FBS; EuroClone), 100 U/mL penicillin, 100 µg/mL streptomycin (both from EuroClone), and 2 mM L-glutamine (EuroClone). Cells were cultured at 37 °C in a humidified incubator with 5% CO_2_. Mycoplasma contamination was routinely checked using the MycoBlue Mycoplasma Detector (Vazyme Biotech Co., Ltd., Nanjing, China).

AlamarBlue™ reagent was purchased from Bio-Rad Laboratories, Inc. (Hercules, CA, USA). Fluorescence measurements were performed using a TECAN SPARK spectrophotometer (TECAN Group Ltd., Männedorf, Switzerland). Dimethyl sulfoxide (DMSO) was purchased from Carlo Erba Reagents S.A.S. (Milan, Italy). Lipopolysaccharide (LPS) from *Salmonella abortus equi* S-form (TLRGRADE®) was purchased from Enzo Life Sciences (Catalog number ALX-581-009, Farmingdale, NY, USA). RNA was extracted using PUREzol reagent (Bio-Rad Laboratories, Inc., Hercules, CA, USA), and RNA purity was assessed using a NanoDrop spectrophotometer (Thermo Fisher Scientific, Madison, WI, USA). The High-Capacity cDNA Reverse Transcription Kit was purchased from Thermo Fisher Scientific. SsoAdvanced Universal SYBR Green Supermix and the CFX96 thermocycler were purchased from Bio-Rad Laboratories, Inc. Primer sets for all amplicons were designed using the IDT Primer Quest Tool (Integrated DNA Technologies, IDTNA).

**1.4 Plant material**

*Cannabis sativa* inflorescences have been supplied by Canvasalus S.r.l. (Monselice, PD, Italy): *C. sativa* III chemotype CBD rich (Cs-CBD05/2022 and Cs-Carmagnola20), *C. sativa* IV chemotype CBG rich (Cs-CBG12/2022), *C. sativa* chemotype CBC rich (Cs-CBC01/2023), *C. sativa* V chemotype (Cs-nocann02/2022). Vouchers specimens of vegetable material are stored in Novara Laboratories.

**1.5 Extraction of cannabis inflorescences**

Ethanolic extracts were obtained from 100 g of each chemotype inflorescences (homogenized hemp). The samples were extracted with ethanol (inflorescences/ethanol ratio 1:5 w/v, 2 x 12 h) in a macerator at room temperature. After the extraction, the vegetal material was removed by filtration using Whatman No 42-filter paper. The solvent was evaporated at reduced pressure to obtain each crude extract (Table 1).

The extraction yield was expressed in % as:

(g of ethanolic extract) / (g of homogenized hemp) * 100

**1.6 Cannabinoids isolation**

*C. sativa* inflorescences (57 g) belonging to chemotype III (voucher specimen Cs-Carmagnola20) were extracted with acetone (inflorescences/acetone ratio 1:10 w/v) in a vertical percolator at room temperature, affording 4 g (7%) of a dark green syrup after evaporation of the solvent at reduced pressure. This was later dissolved at 45 °C in 30 mL of MeOH (with a raw extract/MeOH ratio corresponding to 1:10 w/v) and left at 8 °C to condense fatty acids and waxes. After 12 h, the solution was vacuum filtered with cold MeOH (50 mL) in a sintered funnel protected by a bed of stratified Celite*®*, obtaining 2.9 g of the residual methanolic fraction. This latter portion was filtered through solid-phase extraction on C-18 silica gel (30 g) to remove pigments and unsaturated fatty acids. For this purpose, the fraction was charged on 25 g C-18 silica gel (with a raw extract/stationary phase ratio of 1:10 w/w), packed with MeOH in a sintered funnel (4 × 10 cm) with a side arm for vacuum. Elution with MeOH (100 mL) gave 2.4 g of the purified fraction after evaporation of the solvent at reduced pressure. Once the fraction was dried, it was heated at 130 °C under stirring for 45 °C in a paraffin bath to achieve the decarboxylation followed by TLC (silica PE/EtOAc 70:30 v/v). This latter decarboxylated fraction was fractionated by LPC on silica gel (50 g, PE–EtOAc gradient from 90:10 to 20:80 v/v) to afford three fractions (I, II, and III). Fraction I (745 mg) was further purified with HPLC (250 × 25 mm silica, PE–EtOAc gradient from 90:10 to 80:20 v/v) to afford 180 mg of CBD 1 (Choi et al., 2004) as a white powder, 35 mg of CBC 3 (Claussen et al., 1966) as brownish powder, and 88 mg of CBG 2 (Choi et al., 2004) as a white powder. All the isolated compounds were identified according to ^1^H NMR previously described in the literature. NMR data of the isolated compounds are shown in Fig. S2-S4.

**1.7 Antioxidant activity assays**

The assessment of radical scavenging activity was assessed by different assay: the inhibition of the DPPH^•^ and ABTS^•+^ radicals, and by the Ferric ion antioxidant power (FRAP) following the methodology reported elsewhere (Disca et al., 2024; Jaouhari et al., 2024). For each assay all the crude extracts and pure cannabinoids tested were solubilized in ethanol (1 mg/mL) and opportunely diluted when necessary. All the assays were conducted in triplicate. For DPPH• assay 700 µL of opportunely diluted sample or methanol (control) was added to the same volume of a 100 µmol/L DPPH• methanolic solution. This solution was shaken vigorously and left in the dark at room temperature for 30 min, after which the absorbance was read at 515 nm. Results were expressed as g of Trolox equivalents (TE) per kg of crude extracts through a calibration curve. ABTS+ was prepared by reacting a 7 mmol/L ABTS solution with 2.45 mM potassium persulphate, followed by incubation for 16 h in the dark at room temperature. Prior to initiating the assay, the ABTS solution was diluted with ethanol to an absorbance of 0.700 ± 0.02 at 734 nm. 10 µL of each sample was mixed with 1 mL of ABTS+ solution and it was let react for 6 minutes. Absorbance was read at 734 nm and results were expressed as g of TE per kg of crude extract through a calibration curve. Finally, for the FRAP assay the reactive solution was freshly prepared with 50 mL of 300 mmol/L acetate buffer (pH 3.6), 5 mL of 10 mmol/L 2,4,6-tripyridyl-s-triazine in 40 mmol/L HCl and 5 mL 20 mmol/L FeCl_3_·6H2O in distilled water. Samples (50 µL) or opportunely diluted samples were mixed with 1.5 mL of the FRAP reactive solution. Absorbance was read at 593 nm after 6 minutes and results were expressed as g of TE per kg of crude extract through a calibration curve.

**1.8 Quantitative analysis of cannabinoids and non-cannabinoid phenolic compounds with UHPLC-HRMS**

Phytochemical investigation of samples occurred by UHPLC-HRMS following the method described by Benes et al., (2024). The chromatographic separation was performed using a Acquity [UPLC](https://www.sciencedirect.com/topics/chemistry/ultra-performance-liquid-chromatography) BEH C18 (100 × 2.1 mm; 1.7 μm, Waters, USA) column maintained at 70 °C. The mobile phase A was made of water and MeOH (95:5, v/v) with 5 mM ammonium formate and 0.1% formic acid all UHPLC grade while mobile phase B was isopropyl alcohol, MeOH and water (65:30:5, v/v/v) with 5 mM ammonium formate and 0.1% formic acid all UHPLC grade at the flow rate of 0.300 mL/min. The total run time was 16 minutes with a 3 μL injection volume and gradient elution: 5% B, increase to 60% B by 1 minute, increase to 70% B by 11 minutes, rapid increase to 100% B in 0.5 minutes, isocratic elution for 2 minutes and return to initial conditions for 2.5 minutes. Cannabinoids and non-cannabinoid phenolic compounds were identified by orbital trap mass spectrometer with the following ESI± parameters: sheath/aux gas (N_2_) flow of 45/10 arb. U., aux gas temperature of 300 °C, spray voltage of 3.5 kV and S-lens RF level of 55. The mass spectrometer operated in Full scan mode: 70,000 FWHM resolution, 100–1,000 m/z scan range, AGC target 2e5, maxIT 50 ms. Identification and quantification were carried out utilizing Xcalibur™ 4.0 with each relative standard compound and calibration curve (39 cannabinoids, 12 flavonoids, the dihydrostilbenoid canniprene and the dihydrophenanthrene 5-methoxy-dihydrodenbinobin). Figure of the XICs are shown in Figure S1. The limits of quantification (LOQs), representing the lowest points of calibration, were 0.50–1 mg/kg. The calibration curves were linear up to 50 mg/kg (R² ≥ 0.999). The measurement uncertainty, expressed as relative standard deviation (RSD), was 6–12%.

**1.9 MICs determination**

The antibacterial activity of CS extracts (CS1, CS2, CS3, CS4) pure cannabinoids (CBD, CBG, CBC) and antibiotics (tetracycline, ciprofloxacin, linezolid, methicillin) was evaluated in 96-well microtiter plates by the broth microdilution method described by Wiegand et al., (2008) with minor changes. Stock solutions of CS extracts (20 mg/mL in DMSO) and pure cannabinoids (2 mg/mL in DMSO) were diluted in sterile deionized water to obtain the desired working concentrations. Afterward, bacterial suspensions (~5 × 10^5^ Colony Forming Unit per mL—CFU/mL) in MHB (*S. aureus*, MRSA, *S. epidermidis*, *B. cereus*, *E. coli* and *S. enterica*) or TSB (*L. monocytogenes*) were mixed with CS extracts (final concentrations from 1.25 to 100 µg/mL) and pure compounds (final concentrations from 1.25 to 10 µg/mL) (test wells), antibiotics (used as negative control of growth, final concentrations: tetracycline from 0.25 to 2 µg/mL; ciprofloxacin from 0.063 to 1 µg/mL; linezolid from 0.25 to 2 µg/mL; methicillin from 0.5 to 16 µg/mL), or 0.5% v/v DMSO (used as a positive control for growth) and incubated for 16–20 h at 37 °C.

Furthermore, blank wells containing cannabinoid solutions and sterile growth medium were prepared and incubated under the same conditions to monitor for any aggregation, precipitation, or turbidity due solely to the compounds. The MIC was defined as the lowest concentration of the compound that inhibited visible bacterial growth, as observed with the unaided eye, by comparing each well with the positive control of growth and blank controls. The assays were performed in triplicate and repeated in three independent experiments (n = 9).

**1.10 MBCs determination**

The MBCs of CS extracts (CS1, CS2, CS3, CS4) were determined as described by Mohammad et al., (2017)*.* Briefly, 20-µL aliquots from wells showing no visible growth (from the MIC up to the highest concentration tested) in the 96-well microtiter plate (where the MIC was determined) were transferred onto MHA or TSA plates and incubated at 37 °C for 24 h.

The MBC was defined as the lowest concentration of the compound that killed 99.9% of the bacterial population. Assays were conducted in duplicate and repeated in two independent experiments (n = 4).

**1.11 Time-kill assay**

The time-kill assay was performed in 96-well microtiter plates for CS extracts (CS1, CS2, CS3, CS4) and control antibiotic against MRSA ATCC 43300, following the method described by Brunelli et al., (2023). Stock solutions of CS extracts (4 mg/mL in DMSO) were diluted in sterile deionized water to obtain the desired working solutions. Afterwards, bacterial suspensions (~2 × 10^6^ CFU/mL) in MHB were mixed with CS extracts (final concentration 4 × MIC, test wells), ciprofloxacin (final concentration 4 × MIC, used as bactericidal agent), or 0.5% v/v DMSO (positive control for growth) for 24 h at 37 °C. Aliquots (20 μL) of each sample were collected at 0, 2, 4, 6, 8, 18 and 24 h, serially diluted in 0.9% w/v NaCl, plated onto MHA, and incubated at 37 °C for 18 h before colony counting.

**1.12 Cell viability**

Cell viability was assessed using AlamarBlue™ reagent (Bio-Rad), according to manufacturer's instructions. Briefly, cells were seeded at 1 × 10⁵ cells/well in 96-well plates and incubated overnight. Cells were treated with increasing concentrations of CS extracts (20-100 µg/mL) or pure CBD (25-100 µM). Following 4h treatment, culture medium was replaced with fresh medium containing 10% (v/v) AlamarBlue™ reagent. After 3 h of incubation at 37 °C (protected from light), fluorescence was measured using a SPARK TECAN microplate reader (TECAN) with an excitation wavelength of 530–560 nm and an emission wavelength of 590 nm. All assays were performed in triplicate and repeated in three independent biological experiments. Cell viability was calculated in comparison to vehicle (DMSO)-treated controls.

**1.13 Cell treatments**

RAW 264.7 cells were seeded at 4 × 10⁶ cells/well in 6-well plates and incubated overnight. Cells were pre-treated for 30 minutes with the highest non-cytotoxic concentration of CS extracts (20 µg/mL for CS1, 60 µg/mL for CS2, 40 µg/ml for CS3 and 100 µg/mL for CS4) or vehicle (0,5% v/v). After pre-treatment, cells were stimulated with 100 ng/mL LPS (Salmonella abortus equi S-form; Enzo Life Sciences) for 4 h. Untreated cells were used as control. Cells were lysed with PUREzol reagent (Bio-Rad) for downstream RNA extraction and gene expression analysis.

**1.14 Gene expression analysis**

Total RNA was isolated using PUREzol reagent (Bio-Rad) following the manufacturer’s protocol. Briefly, cells were homogenized in PUREzol, and RNA was phase-separated with chloroform, precipitated with isopropanol, and washed twice with 75% ethanol. The RNA pellet was air-dried and resuspended in RNase-free water. RNA concentration and purity was assessed using a Nanodrop spectrophotometer (Thermo Fisher Scientific). Only samples with a 260/280 and a 260/230 OD ratios > 1.5 were processed further. 2 μg of total RNA was reverse-transcribed using the High-Capacity cDNA Reverse Transcription Kit (Thermo Fisher Scientific) according to the manufacturer’s instructions. Quantitative PCR (qPCR) was performed in triplicate using SsoAdvanced Universal SYBR Green Supermix (Bio-Rad) on a CFX96 thermocycler (Bio-Rad). Gene-specific primers (Table S1) were used for amplification. Inflammatory gene expression levels were normalized to GAPDH as the housekeeping gene and calculated using the 2^(−ΔΔCt) method**.** Results are shown as fold change relative to untreated cells (controls)**.**

**1.15 Statistical analysis**

All statistical analyses were performed using R software (version 4.3.1; Boston, USA) or GraphPad Prism (version 10.5.0; San Diego, CA, USA). Results are presented as mean ± standard deviation (SD) from three independent experiments. Differences between groups were assessed by analysis of variance (ANOVA), followed by Tukey’s honest significant difference (HSD) test. A p-value of <0.05 was considered statistically significant.

Table S1. Primer sequences used for qPCR

| Gene | Forward | Reverse |
| --- | --- | --- |
| *Gapdh* | TTCAACGGCACAGTCAAG | CCAGTAGACTCCACGACATA |
| *Il1b* | AAGTTGACGGACCCCAAAAGAT | TGTTGATGTGCTGCTGCGA |
| *Il6* | GGATACCACTCCCAACAGACCT | GCCATTGCACAACTCTTTTCTC |
| *Cox2*  *Il10*  *Il1ra* | AATGAGTACCGCAAACGCTTC  GCAGGACTTTAAGGGTTACTTGG  TGGCCTAATCCCCATGATGA | CAGCCATTTCCTTCTCTCCTGTA  GGGGCATCACTTCTACCAGG  AGACTTCACCCAGATGGCAGAG |

Table S2. Cannabinoids content expressed as mg/kg of crude extracts.

| Compound (mg/kg) | CS1 | CS2 | CS3 | CS4 |  |
| --- | --- | --- | --- | --- | --- |
| CBGO | 5.45 ± 0.65 | 5.78 ± 0.52 | <LOQ | <LOQ |  |
| CBGOA | 4.94 ± 0.27 | 13.59 ± 0.05 | 5.22 ± 0.15 | <LOQ |  |
| CBDVA | 430.16 ± 11.05 | 21.61 ± 0.84 | 30.56 ± 4.51 | 7.72 ± 0.29 |  |
| CBND | 570.42 ± 35.63 | <LOQ | <LOQ | <LOQ |  |
| CBDV | 3662.9 ± 325.29 | 17.5 ± 1.98 | 10.25 ± 0.17 | 8.3 ± 1.21 |  |
| CBGV | 39.31 ± 3.27 | 214.21 ± 16.13 | 42.41 ± 0.26 | <LOQ |  |
| CBGVA | <LOQ | 90.66 ± 1.01 | 129.92 ± 5.54 | <LOQ |  |
| CBDB | 826.26 ± 32.94 | <LOQ | <LOQ | <LOQ |  |
| CBGB | <LOQ | 150.44 ± 11.09 | 58.22 ± 7.63 | <LOQ |  |
| CBE | 4043.57 ± 56.14 | <LOQ | <LOQ | <LOQ |  |
| CBCO | 52.41 ± 0.41 | 70.58 ± 4.49 | 45.89 ± 1.42 | <LOQ |  |
| CBV | 67.59 ± 4 | 41.3 ± 0.29 | 14.75 ± 0.83 | <LOQ |  |
| CBDA | 37962.57 ± 3056.04 | 277.48 ± 34.36 | 7651.57 ± 91.19 | 538.68 ± 93.88 |  |
| CBG | 5214.83 ± 305.09 | 178549.01 ± 7133.88 | 23461.56 ± 301.19 | <LOQ |  |
| CBD | 360585.68 ± 51718.52 | 1041.7 ± 476.7 | 3262.66 ± 455.92 | 45.99 ± 0.97 |  |
| CBVA | 14.02 ± 0.16 | 5.69 ± 0.09 | 4.76 ± 0.44 | <LOQ |  |
| CBGA | 3730.22 ± 9.03 | 89506.85 ± 1455.08 | 37615.99 ± 31.21 | 21.55 ± 1.81 |  |
| THCV | 20.68 ± 1.17 | 20.19 ± 1.49 | 129.94 ± 18.86 | <LOQ |  |
| CBDH | 50.32 ± 0.86 | <LOQ | <LOQ | <LOQ |  |
| CBCV | 209.47 ± 4.77 | 162.85 ± 28.37 | 1831.96 ± 62.49 | <LOQ |  |
| THCVA | <LOQ | <LOQ | 5.13 ± 0.16 | <LOQ |  |
| CBN | 6209.2 ± 396.78 | 1622.12 ± 83.3 | 498.86 ± 20.04 | <LOQ |  |
| CBCVA | 40.38 ± 1.06 | 16.03 ± 0.03 | 196.94 ± 12.67 | <LOQ |  |
| cis-delta-THC | 2684.71 ± 102.8 | 574.42 ± 31.78 | 1313.87 ± 28.59 | <LOQ |  |
| CBDP | 38.1 ± 2.92 | <LOQ | <LOQ | <LOQ |  |
| CBNA | 138.18 ± 1.99 | 35.49 ± 2.05 | 45.91 ± 3.21 | 4.03 ± 0.35 |  |
| THC | 1941.55 ± 66.2 | 880.67 ± 194.45 | 3660.25 ± 619.28 | <LOQ |  |
| CBL | 958.4 ± 79.29 | 682.92 ± 68.76 | 2373.58 ± 30.34 | <LOQ |  |
| CBC | 11326.08 ± 40.21 | 30303.66 ± 1074.21 | 250985.99 ± 17657.37 | 31.71 ± 0.72 |  |
| THCA | 155.47 ± 14.28 | 39.64 ± 0.86 | 255.45 ± 6.21 | 35.87 ± 8.95 |  |
| CBCA | 1426.08 ± 9.51 | 2683.93 ± 43.62 | 29130.89 ± 451.62 | 29.65 ± 1.59 |  |
| CBTC | 5194.69 ± 287.94 | 7312.18 ± 119.54 | 57305.66 ± 1265.81 | 6.49 ± 0.06 |  |
| CBLA | 227.89 ± 15.84 | 195.66 ± 24.98 | 1355.32 ± 136.07 | 5.89 ± 0.38 |  |
| Total | 447831 | 314536 | 421423 | 736 |  |

Different letters indicate statistical differences (p < 0.05), within the same molecule.

Table S3. Flavonoids content expressed as mg/kg of crude extracts. Different letters indicate statistical differences (p>0.05) within the same molecule.

| Compound (mg/kg) | CS1 | CS2 | CS3 | CS4 |
| --- | --- | --- | --- | --- |
| Vitexin | < LOQ | < LOQ | < LOQ | 75.05 ± 8.03 |
| Luteolin | 64.36 ± 8.03^c^ | 26.01 ± 8.59^d^ | 231.81 ± 6.3^a^ | 129.53 ± 7.11^b^ |
| Quercetin |  | 15.08 ± 1.87 |  |  |
| Apigenin | 171.23 ± 1.62^c^ | 63.1 ± 8.57^d^ | 462.62 ± 2.05^b^ | 599.98 ± 24.19^a^ |
| Chrysoeriol | 117.5 ± 13.41b^c^ | 8.64 ± 0.47^c^ | 254.39 ± 17.23^b^ | 1711 ± 70.71^a^ |
| Cannflavin B | 1468.34 ± 60.15^b^ | 355.55 ± 10.85^c^ | 2899.43 ± 160.05^a^ | 2965.83 ± 91.02^a^ |
| Cannflavin A | 2557.6 ± 91.14^c^ | 1892.29 ± 66.84^d^ | 7709.13 ± 62.28^b^ | 10802.84 ± 12.55^a^ |
| Morin | 2.96 ± 0.54 | 4.49 ± 0.66 |  | 2.83 ± 0.1 |
| Diosmetin | 93.79 ± 10.73c | 6.56 ± 0.45^d^ | 217.91 ± 1.47^b^ | 826.52 ± 12.81^a^ |
| Baicalein | 18.92 ± 2.55^c^ | 2 ± 0.23^d^ | 62.25 ± 0.7^b^ | 86.22 ± 2.96^a^ |
| Genistein | 11.96 ± 1.35^c^ | 1.29 ± 0.04^d^ | 38.68 ± 1.36^b^ | 51.55 ± 0.97^a^ |
| Canniprene | 502 ± 21^b^ | < LOQ | 4168 ± 337^a^ | 491 ± 16^b^ |
| 5-methoxy-dihydrodenbinobene | < LOQ | < LOQ | 11168 ± 74^a^ | 940 ± 108^b^ |
| Total | 5008^c^ | 2375^d^ | 27212^a^ | 18682^b^ |

Different letters indicate statistical differences (p < 0.05), within the same molecule.

Fig. S1: XICs of all standard cannabinoids and flavonoids detected (100 ng/mL)


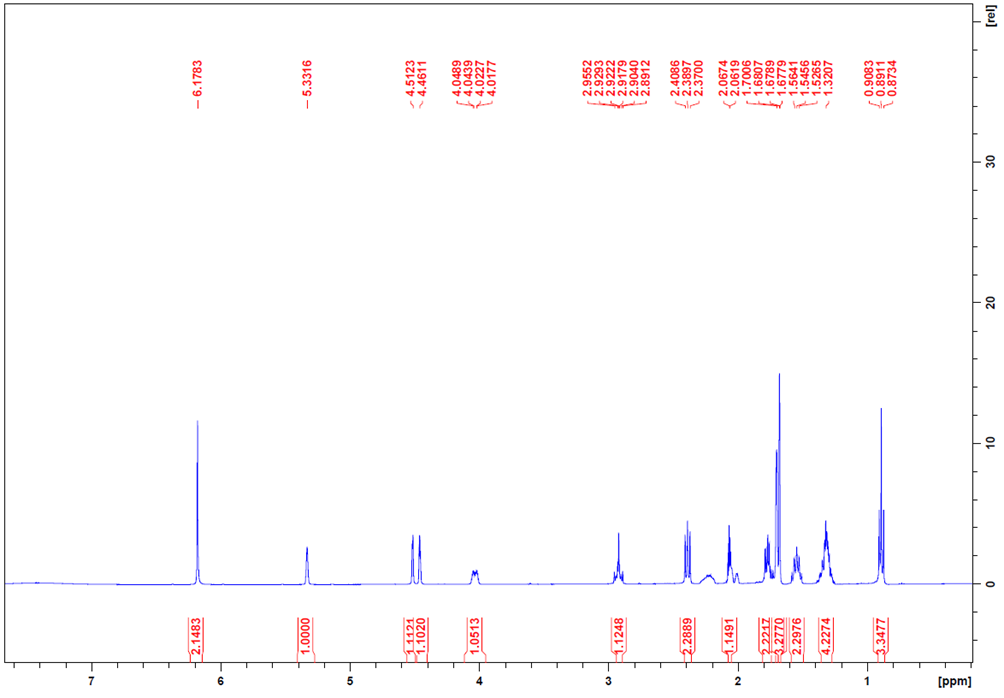


Fig. S2: ^1^H NMR (400 MHz) of cannabidiol (CBD) in CDCl_3_


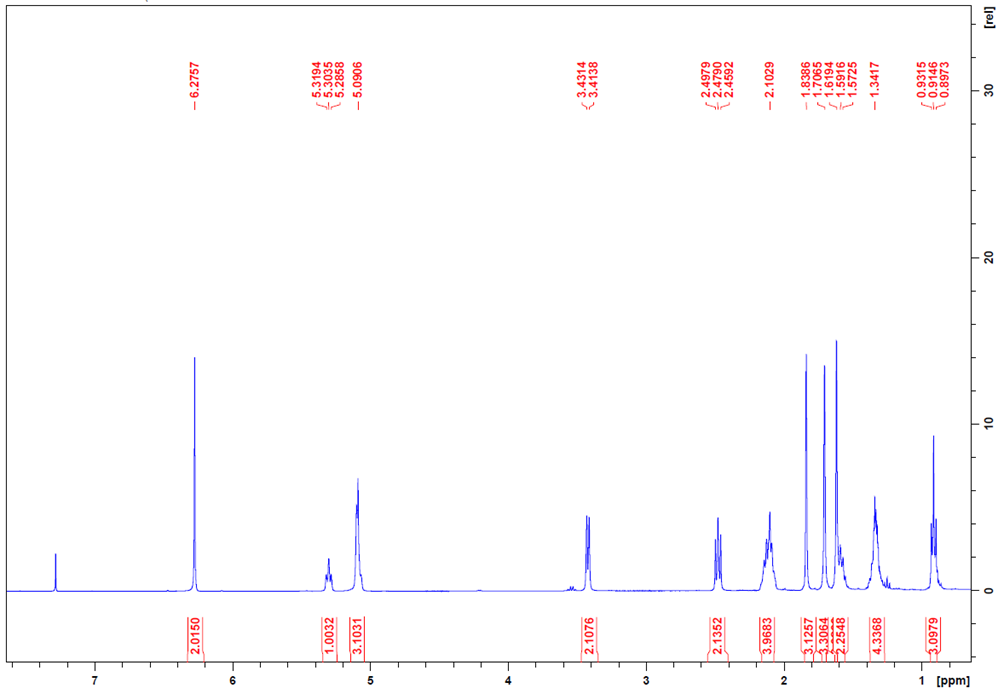
Fig. Figure S3: ^1^H NMR (400 MHz) of cannabigerol (CBG) in CDCl_3_


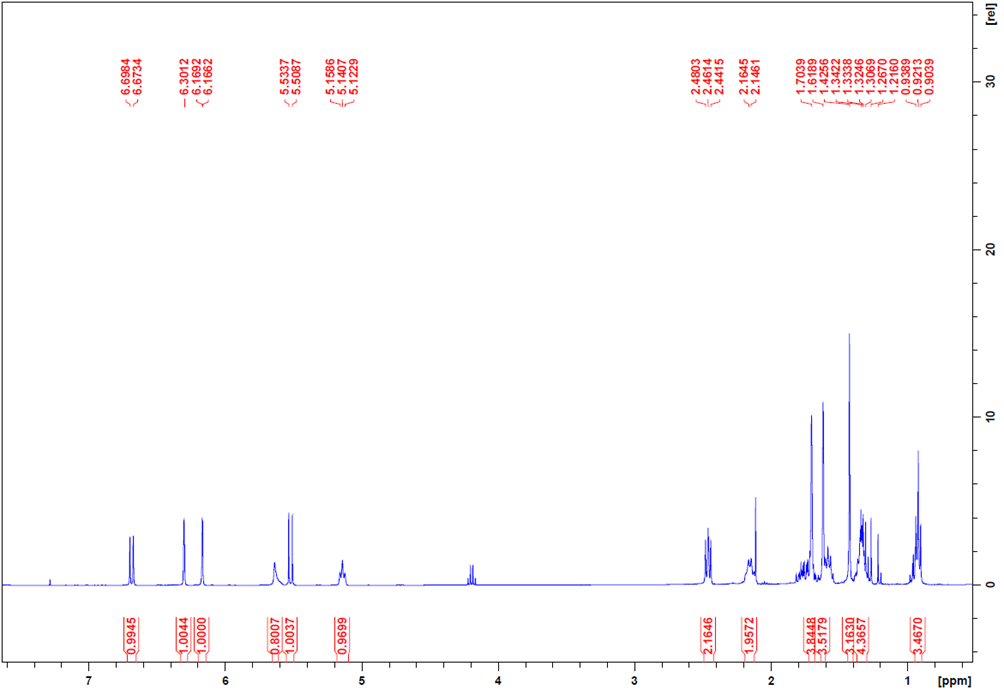


Fig. S4: ^1^H NMR (400 MHz) of cannabichromene (CBC) in CDCl_3_

**2. REFERENCES**

Benes, F., Binova, Z., Zlechovcova, M., Maly, M., Stranska, M., & Hajslova, J. (2024). Thermally induced changes in the profiles of phytocannabinoids and other bioactive compounds in *Cannabis sativa* L. inflorescences. *Food Research International*, *190*, 114487. https://doi.org/10.1016/j.foodres.2024.114487

Brunelli, F., Ceresa, C., Aprile, S., Coppo, L., Castiglioni, B., Bosetti, M., Fracchia, L., & Tron, G. C. (2023). Isocyanides in med chem: A scaffold hopping approach for the identification of novel 4-isocyanophenylamides as potent antibacterial agents against methicillin-resistant *Staphylococcus* *aureus*. *European Journal of Medicinal Chemistry*, *246*, 114950. https://doi.org/10.1016/j.ejmech.2022.114950

Disca, V., Jaouhari, Y., Carrà, F., Martoccia, M., Travaglia, F., Locatelli, M., Bordiga, M., & Arlorio, M. (2024). Effect of Carbohydrase Treatment on the Dietary Fibers and Bioactive Compounds of Cocoa Bean Shells (CBSs). *Foods*, *13*(16), Article 16. https://doi.org/10.3390/foods13162545

Jaouhari, Y., Disca, V., Ferreira-Santos, P., Alvaredo-López-Vizcaíno, A., Travaglia, F., Bordiga, M., & Locatelli, M. (2024). Valorization of Date Fruit (Phoenix dactylifera L.) as a Potential Functional Food and Ingredient: Characterization of Fiber, Oligosaccharides, and Antioxidant Polyphenols. *Molecules*, *29*(19), Article 19. https://doi.org/10.3390/molecules29194606

Mohammad, H., Younis, W., Chen, L., Peters, C. E., Pogliano, J., Pogliano, K., Cooper, B., Zhang, J., Mayhoub, A., Oldfield, E., Cushman, M., & Seleem, M. N. (2017). Phenylthiazole Antibacterial Agents Targeting Cell Wall Synthesis Exhibit Potent Activity in Vitro and in Vivo against Vancomycin-Resistant Enterococci. *Journal of Medicinal Chemistry*, *60*(6), 2425–2438. https://doi.org/10.1021/acs.jmedchem.6b01780

Wiegand, I., Hilpert, K., & Hancock, R. E. W. (2008). Agar and broth dilution methods to determine the minimal inhibitory concentration (MIC) of antimicrobial substances. *Nature Protocols*, *3*(2), 163–175. https://doi.org/10.1038/nprot.2007.521
